# Physical therapy and deep brain stimulation in Parkinson’s Disease: protocol for a pilot randomized controlled trial

**DOI:** 10.1186/s40814-018-0243-2

**Published:** 2018-02-21

**Authors:** Ryan P. Duncan, Linda R. Van Dillen, Jane M. Garbutt, Gammon M. Earhart, Joel S. Perlmutter

**Affiliations:** 1 Program in Physical Therapy, Washington University School of Medicine in Saint Louis, Campus Box 8502, 4444 Forest Park Blvd, St. Louis, MO 63108 USA; 2 Department of Neurology, Washington University School of Medicine in Saint Louis, St. Louis, MO USA; 3 Department of Orthopaedic Surgery, Washington University School of Medicine in Saint Louis, St. Louis, MO USA; 4 Department of Medicine, Washington University School of Medicine in Saint Louis, St. Louis, MO USA; 5 Department of Pediatrics, Washington University School of Medicine in Saint Louis, St. Louis, MO USA; 6 Department of Neuroscience, Washington University School of Medicine in Saint Louis, St. Louis, MO USA; 7 Department of Radiology, Washington University School of Medicine in Saint Louis, St. Louis, MO USA; 8 Program in Occupational Therapy, Washington University School of Medicine in Saint Louis, St. Louis, MO USA

**Keywords:** Parkinson’s disease, Deep brain stimulation, Physical therapy, Balance, Gait

## Abstract

**Background:**

Subthalamic nucleus deep brain stimulation (STN-DBS) reduces tremor, muscle stiffness, and bradykinesia in people with Parkinson’s Disease (PD). Walking speed, known to be reduced in PD, typically improves after surgery; however, other important aspects of gait may not improve. Furthermore, balance may worsen and falls may increase after STN-DBS. Thus, interventions to improve balance and gait could reduce morbidity and improve quality of life following STN-DBS. Physical therapy (PT) effectively improves balance and gait in people with PD, but studies on the effects of PT have not been extended to those treated with STN-DBS. As such, the efficacy, safety, and feasibility of PT in this population remain to be determined. The purpose of this pilot study is to address these unmet needs. We hypothesize that PT designed to target balance and gait impairment will be effective, safe, and feasible in this population.

**Methods/design:**

Participants with PD treated with STN-DBS will be randomly assigned to either a PT or control group. Participants assigned to PT will complete an 8-week, twice-weekly PT program consisting of exercises designed to improve balance and gait. Control group participants will receive the current standard of care following STN-DBS, which does not include prescription of PT. The primary aim is to assess preliminary efficacy of PT on balance (Balance Evaluation Systems Test). A secondary aim is to assess efficacy of PT on gait (GAITRite instrumented walkway). Participants will be assessed OFF medication/OFF stimulation and ON medication/ON stimulation at baseline and at 8 and 12 weeks after baseline. Adverse events will be measured over the duration of the study, and adherence to PT will be measured to determine feasibility.

**Discussion:**

To our knowledge, this will be the first study to explore the preliminary efficacy, safety, and feasibility of PT for individuals with PD with STN-DBS. If the study suggests potential efficacy, then this would justify larger trials to test effectiveness and safety of PT for those with PD with STN-DBS.

**Trial registration:**

NCT03181282 (clinicaltrials.gov). Registered on 7 June 2017.

**Electronic supplementary material:**

The online version of this article (10.1186/s40814-018-0243-2) contains supplementary material, which is available to authorized users.

## Background

Subthalamic nucleus deep brain stimulation (STN-DBS) effectively reduces tremor, rigidity, and bradykinesia in people with Parkinson’s Disease (PD) [1]. The annual number of STN-DBS procedures for PD totals between 8000 and 10,000 [2] and will likely rise in the near future [3]. Despite the spread of this new treatment modality, the effect on postural instability and gait is unclear. Postural stability initially improves following STN-DBS [4–6]; however, these benefits may not endure. For example, postural responses worsened in individuals 6 months post-DBS surgery [7], and STN-DBS did not improve balance in individuals with PD who had abnormal quiet stance prior to surgery [8]. Further, STN-DBS initially improves postural instability and gait difficulty (PIGD), but 2 years after surgery, these deficits were worse than before surgery [9]. Also, falls may increase after STN-DBS surgery [10]. STN-DBS effectively improves gait spatial parameters (e.g., stride length), but may not change temporal parameters. Stride-to-stride variability, known to be impaired [11] in PD, did not change following STN-DBS [12]. More importantly, approximately 42% of individuals post-STN-DBS subjectively reported worsened gait [13]. Deficits in balance and gait may lead to falls, fall-related complications, and physical inactivity in people with PD [14]. These negative effects may lead to a “malignant” form of PD with reduced quality of life and increased mortality [14].

Physical therapy (PT) effectively complements pharmacologic interventions to improve postural stability and gait in people with PD who do not have DBS [15–18]. Given the potential for worsening of balance and gait following STN-DBS, PT may be an effective adjunct treatment to optimize long-term outcomes after STN-DBS surgery. However, no studies to date have assessed the impact of PT for those with PD who have STN-DBS as these individuals are frequently excluded from exercise trials [18–23]. Although the current standard of care permits return to exercise within weeks following STN-DBS, there is no explicit call for PT [24]. As such, the efficacy, safety, and feasibility of exercise in this population remain to be determined.

The primary purpose of this study is to examine the preliminary efficacy of PT on balance in people with PD with STN-DBS. We chose balance as the primary outcome because balance is most likely to remain impaired after surgery [7, 8]. Secondarily, we will examine the preliminary efficacy of PT on gait as well as safety and feasibility of the intervention in this population in people with PD with STN-DBS. We hypothesize that participants assigned to PT will 1) demonstrate improvements in balance and gait while controls will not change, 2) not experience more adverse events than those in the control group, and 3) complete at least 80% of prescribed PT sessions.

## Methods/design

This pilot study is designed as a randomized controlled trial. Participants will be randomly assigned (1:1 ratio) to either the PT intervention group or control group in a consecutive fashion. Randomization will be stratified by the participant’s medication and stimulation condition for their first visit to the laboratory. Using an internet randomization scheme generator (http://www.graphpad.com/quickcalcs/randomize1.cfm), a study team member will write the group assignment number (i.e., 1 = PT, 2 = Control) on a piece of paper wrapped in tin foil. This will be placed in an opaque envelope and sealed. The study team member conducting these procedures will have no role in scheduling participants, data collection, or assigning participants to the intervention. After participants complete both pre-test assessments (see detailed information below), they will be allowed to open the envelope to determine their group assignment.

### Participants

All participants will be at least 30 years of age and meet the following inclusion criteria: 1) neurologist diagnosed idiopathic PD [25–27] between Hoehn and Yahr (H&Y) stages II–IV (measured on stimulation and medication), 2) at least 1-year post-STN-DBS, 3) able to attend assessment sessions, and 4) able to provide informed consent. Participants will be excluded if they have any of the following: 1) diagnosis of atypical parkinsonism, 2) H&Y stages I or V, 3) evidence of dementia (i.e., Mini-Mental Status Exam (MMSE) score ≤ 24/30 [28]), or 4) inability to walk 10 m with or without an assistive device. Written informed consent will be obtained from each participant in accordance with the policies and procedures of the Washington University Human Research Protection Office (approved November 9, 2016; IRB ID: 201609148).

### Intervention

#### PT intervention

Participants assigned to PT will attend a 1-h visit with a physical therapist (RPD) twice weekly for 8 weeks and will also complete an individualized home exercise program twice weekly. The PT intervention, which will mirror traditional PT for those with PD [15], will include exercises designed to improve balance, gait, and lower extremity strength. PT will be provided in the outpatient physical therapy clinical practice at the Washington University Program in Physical Therapy. In addition to the PT intervention, those assigned to the PT group will receive the current standard of care, defined as clinical optimization by the neurologist of STN-DBS parameters and anti-PD medication.

Postural stability exercises will follow the framework provided by Schoneburg and colleagues [29], targeting quiet stance, anticipatory and reactive postural adjustments, and dynamic postural control. Balance exercises following a similar framework improved balance and reduced falls in people with PD *without* STN-DBS [18]. A detailed protocol of the postural stability exercises has been developed to enhance reproducibility and guide decision-making for exercise progression. Exercise performance will be evaluated each visit and progressed according to rules set forth in the PT intervention protocol (see Additional file 1). The starting level for each exercise level will be commensurate with each participant’s ON medication/ON stimulation baseline BESTest performance. Gait exercises will include treadmill walking and dual-task gait training with the goal of improving spatiotemporal gait parameters. Both treadmill walking [30, 31] and dual-task gait training [32, 33] have been used to reduce gait variability and improve spatiotemporal gait parameters in people with PD *without* DBS.

#### Home exercise program

A home exercise program (HEP), to be performed twice weekly, will be prescribed at the participant’s initial visit with the physical therapist. The HEP incorporates the following lower extremity functional movements: sit-to-stand, heel raises, hip flexion, and standing half squats. The HEP is designed to address biomechanical constraints related to balance performance. A detailed HEP protocol will enhance reproducibility and guide exercise progression. Performance of the HEP will be reviewed weekly, and progression will be determined based on participant performance according to rules set forth in the HEP protocol (see Additional file 2).

#### Control group

As in the PT group, participants in the control group will receive the current standard of care (i.e., optimization of STN-DBS parameters and anti-PD medication as needed) following STN-DBS. However, those in the control group will not receive prescribed exercise from a physical therapist.

### Assessments

All participants will undergo the same battery of balance and gait tests. Although STN-DBS settings are typically stable at 12 months post-surgery, participants will follow up with their neurologists as needed for programming and medication adjustments throughout the study. All changes in programming settings and medication dosages will be noted. Participants will be tested in the following conditions: 1) OFF stimulation and OFF medication and 2) ON stimulation and ON medication. Testing participants OFF stimulation/OFF medication will allow us to determine if the addition of PT after STN-DBS affects balance and gait independent of other treatments. OFF medication is defined as greater than or equal to 12 h since the last intake of anti-PD medication. For OFF stimulation/OFF medication testing, participants will arrive at the laboratory OFF medication but with stimulators on. The stimulators will be turned OFF upon arrival at the laboratory and testing will commence 45 min after [34] the stimulators are turned OFF. Testing ON stimulation/ON medication will provide insight into how participants perform on an everyday basis. ON medication is defined as 1–1.5 h after medication intake. For ON stimulation/ON medication assessments, participants will arrive at the laboratory 1–1.5 h after taking their normal anti-PD medication dose with stimulators on and stimulators will remain on throughout the session.

**Fig. 1:**
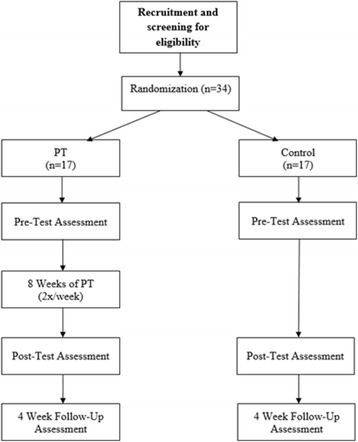
Diagram depicting participant recruitment, randomization, assessments, and intervention

Assessments will occur at baseline and at 8 weeks (i.e., post-test) and 12 weeks after baseline (i.e., 12-week follow up) (Fig. 1). A rater, blinded to group assignment, will collect outcomes at each time point. Assessments will take place on two separate days, and the order of testing condition (OFF medication/OFF stimulation vs. ON medication/ON stimulation) will be randomized. The Movement Disorders Society—Unified Parkinson’s Disease Rating Scale III (MDS-UPDRS III) [35], a gold-standard measure of PD motor severity, will be administered in each condition.

### Primary outcome

#### Efficacy

Preliminary efficacy of the 8-week intervention on balance will be assessed using the 27-item Balance Evaluation Systems Test (BESTest). The BESTest [36] is a clinical assessment of balance that has a total possible score of 108 points, with higher scores indicating better balance. The BESTest is highly reliable [37] in people with PD.

### Secondary outcomes

#### Efficacy

Preliminary efficacy of the intervention on gait will be assessed with a GAITRite (CIR Systems, Sparta, NJ) to measure spatiotemporal gait parameters (i.e., gait velocity, stride length, and stride-to-stride variability). This is a 5-m computerized walkway that contains pressure sensors and maps footfalls during walking. Average gait velocity, stride length, and stride-to-stride variability will be collected over five trials of preferred-pace forward walking.

We also will collect an abbreviated battery of outcomes each week throughout the 8-week PT intervention to determine the response to PT and whether or how this changes over the study period. The battery will consist of the following measures: 10-m gait speed, Timed Up and Go [38], and single-limb stance time.

#### Safety

At the baseline visit, all participants will be fully instructed on what constitutes an adverse event. For this study, an adverse event is defined as “any untoward or unfavorable medical occurrence in a human subject, including any abnormal sign (for example, abnormal physical exam or laboratory finding), symptom, or disease, temporally associated with the subject’s participation in the research, whether or not considered related to the subject’s participation in the research.” [39]. Weekly calls to participants will be conducted by the research coordinator to track any adverse events. Participants will be asked questions related to falls, orthopedic injuries, unscheduled visits to a physician, or hospital admissions. Furthermore, participants will be instructed to call a member of the study team as soon as possible after an adverse event. We will also monitor adverse events directly related to the intervention. Adverse events may or may not be determined as serious. Serious adverse events are defined as unanticipated problems that 1) would involve risks to participants, 2) relate or possibly relate to participation in the research, and 3) suggest the research places participants at greater risk of harm than previously known or recognized. Examples of potential serious adverse events are a fall with serious injury (e.g., fracture and head injury) or a major cardiac event. If the serious adverse event occurs during an intervention session or during performance of the HEP, it will be determined that it is directly related to the intervention. Our a priori threshold for serious adverse events that would constitute not moving forward with this intervention in a larger trial is greater than one serious adverse event.

#### Feasibility

Feasibility will be measured by adherence to the PT intervention and will be assessed in two ways. First, participation in PT sessions will be tracked allowing for calculation of a total percentage of PT sessions attended. Second, adherence to the home exercise program (HEP) will be tracked using a log-book in which participants record a number of sets and repetitions completed for each exercise. This will be reviewed weekly with the physical therapist. To promote adherence, participants who miss a PT session will be phoned immediately after the missed appointment by the research coordinator to remind them of their next appointment and urge them to continue their HEP. Our a priori threshold for adherence to the intervention which would constitute not moving forward with this intervention in a larger trial would be less than an average of 80%. This 80% threshold is the typical adherence rate for short-term exercise programs in PD [40].

We will also measure PD motor severity and quality of life. The MDS-UPDRS III [35], the gold-standard measure of motor sign severity, will be used to determine if PT impacts motor sign severity in people with STN-DBS when compared to the current standard of care. Quality of life will be measured using the Parkinson’s Disease Questionnaire-39 [41].

### Data analysis

For intervention effects on balance and gait, the BESTest total score and all gait variables (i.e., gait velocity, stride length, and stride-to-stride variability) will be analyzed using repeated measures analysis of variance to test for main effects of group (PT, control) and time (baseline, 8 weeks, 12 weeks) and group × time interactions (*α* = 0.05).

For safety, the total number of adverse events will be reported for each group allowing for qualitative comparisons. For feasibility, adherence will be reported for both attendance to PT sessions and HEP completion. Both will be expressed as percent of sessions completed.

### Power analysis

To our knowledge, there are no intervention studies that specifically examined the efficacy of physical therapy on balance in people with PD with STN-DBS. Given that this is a pilot study, the authors chose an effect size that would be meaningful and required to support moving forward with a larger trial. As such, with a medium effect size of 0.25 (Cohen’s *f*) [42] and an alpha level of 0.05, 14 participants per group will provide 82% power to detect significant differences between or within groups for the primary outcome (i.e., BESTest). Factoring in the potential for a 20% attrition rate based on prior studies, we plan to recruit 17 participants per group. Point estimates and variance around the point estimates for the change in balance and gait will be used to power a larger trial if the results support the hypotheses. This pilot study is not powered to detect significant differences in the number of adverse events between groups, which is declared as a limitation.

## Discussion

This pilot study has several strengths. First, the randomized design helps to control for the effect of potential unmeasured confounders on the outcomes of interest. Second, blinded assessments will control for potential rater bias. Third, testing people OFF medication/OFF stimulation allows for determination of change after the intervention in the absence of optimal medical and surgical management. Testing participants ON medication/ON stimulation allows for determination of change in motor performance in the setting of optimal pharmacological and surgical management. Finally, the detailed intervention protocol (See Additional files 1 and 2) delivered by a single physical therapist will enhance standardization, reproducibility, and treatment fidelity. Historically, studies of physical interventions in PD lack the necessary information to accurately reproduce the treatment. It is our hope that the depth of detail and decision algorithm provided will reduce the ambiguity encountered when attempting to determine how and when it was decided that treatment would be progressed.

While this study has several strengths, some weaknesses exist. First, the proposed small sample size limits the ability to generalize our results to a larger population. However, because this is a pilot study, the results may provide the preliminary data to justify a larger randomized controlled trial. Second, people less than 1-year post-STN-DBS will be excluded to minimize the potential for changes in DBS programming. If the study results support our hypotheses, future studies could evaluate the efficacy of PT for balance and gait deficits before and/or immediately post-surgery, increasing the potential impact of this research. Finally, only those with STN-DBS will be included to maximize sample homogeneity in this pilot trial. In the future, investigators also may study those with DBS in other brain locations (e.g., globus pallidus internus).

As PT is not currently part of the standard of care for those with STN-DBS, the results, if in support of the hypotheses, may lend initial support to the addition of PT into the standard plan of care for these individuals. Additionally, this preliminary work has the potential to generate many future research questions. Data from this study could be used to further explore how parameters of exercise dose (i.e., intensity, frequency, duration) can be best modified to optimize mobility-related outcomes in those with STN-DBS. If our results indicate efficacy of PT in this population, investigators may study the benefits of PT prior to STN-DBS and determine if PT modifies the trajectory of motor disability over time. In sum, this work represents the start of a line of research that could challenge current clinical practice paradigms in the management of those with PD with STN-DBS. Physical therapy is a personalized intervention that may be useful to address specific movement impairments that remain even when patients are on optimal regimens of medication and STN-DBS.

### Data monitoring

A Data and Safety Monitoring Board has not been formally established as the intervention is considered to be low risk. All adverse events will be reported to the principal investigator (RPD). These will be reviewed on a monthly basis with the study team (RPD, LVD, JSP, GME, and JG) to determine if adverse events are or are not related to the intervention and whether the trial should be discontinued on the basis of safety problems.

### Trial status

This trial was registered on clinicaltrials.gov (NCT: 03181282) on June 7, 2017. Recruitment of participants was initiated on July 12, 2017. Accounting for attrition, 34 participants (17 per group) will be recruited to participate in the study. With an expected inclusion rate of three to four participants per month, an estimated recruitment period of 12 months is required beginning from the start of recruitment.

### Dissemination of results

The study results will be shared through a peer-reviewed manuscript and through conference presentations.

## Additional files

Additional file 1: Physical therapy for deep brain stimulation: in-clinic treatment program. (DOCX 35 kb)

Additional file 2: Physical therapy for deep brain stimulation: home exercise program. (DOCX 14 kb)

## Abbreviations

BESTest: Balance Evaluation Systems Test
H&Y: Hoehn and Yahr
HEP: Home exercise program
MDS-UPDRS III: Movement Disorder Society—Unified Parkinson’s Disease Rating Scale Subsection III
MMSE: Mini-Mental Status Exam
PD: Parkinson’s disease
PIGD: Postural instability gait difficulty
PT: Physical therapy
STN-DBS: Subthalamic nucleus deep brain stimulation
