# Supplementary material for: Physical therapy and deep brain stimulation in Parkinson’s Disease: protocol for a pilot randomized controlled trial
Source: Pilot Feasibility Stud. 2018 Feb 21;4:54. doi: 10.1186/s40814-018-0243-2 (PMC5822622; doi:10.1186/s40814-018-0243-2)
Supplement: Supplementary file 1 — Physical therapy for deep brain stimulation: in-clinic treatment program. (DOCX 35 kb) [file 40814_2018_243_MOESM1_ESM.docx]

**Physical Therapy for Deep Brain Stimulation**

**In-Clinic Treatment Program**

**Ryan Duncan, PT, DPT**

**Table of Contents** **Page(s)**

**Stability Limits / Verticality Exercises**

Forward Reaching Levels 1-8 3-10

Lateral Reaching Levels 1-8 11-18

**Anticipatory Postural Adjustments**

Multi-Directional Stepping: Levels 1-8 19-27

Step Up and Over Obstacle: Levels 1-8 28-35

**Postural Responses**

Perturbation Training: Level 1 36

**Sensory Orientation**

Semi-Tandem/Tandem Stance: Levels 1-11 37-46

**Stability in Gait**

Treadmill Walking: Week 1, Visit 1 to Week 8, Visit 2 47-62

Dual (Motor) Task Walking: Week 1, Visit 1 to Week 8, Visit 2 63-78

Dual (Cognitive) Task Walking: Week 1, Visit 1 to Week 8, Visit 2 78-94

**Stability Limits / Verticality Exercises**

**Forward Reaching for Object**

**Level 1:** Forward Reaching for Object Without UE Support

**Equipment:** Table, cup

Instructions: I am going to ask you to reach for the cup and hand it to me while maintaining your balance. Stand with your feet shoulder-width apart. Reach for the object while trying to not to let your heels rise from the ground. Once you grab the cup, hand it to me. We will repeat this reaching exercise 10 times.

****Object should be set a distance that requires the participant to reach as far as possible without allowing heels to come off the surface.****

**Verbal Cues for Feedback:**

- Bend at your hips while reaching.
- Allow your weight to shift toward the front of your feet as you reach.
- Keep your heels on the ground throughout the task.

**Successful Repetition:**

- Completes 10 reaches successfully without loss of balance (i.e. stepping strategy).
- Maintains heels on the ground for all 10 repetitions.

**Unsuccessful Repetition:**

- Unable to complete 10 repetitions without loss of balance (i.e. stepping strategy).
- Unable to maintain heels on the ground while reaching.

**Performance**

- **Set 1**
  - Successful Repetitions: ______ /10
  - Number of Verbal Cues Given: ______________
- **Set 2**
  - Successful Repetitions: ______ /10
  - Number of Verbal Cues Given: ______________
- **Set 3**
  - Successful Repetitions: ______ /10
  - Number of Verbal Cues Given: ______________

**Criteria for Progression**

- Successful performance of all repetitions and sets.
- No more than 2 verbal cues provided per set.

**Does the participant meet the criteria for progression? YES / NO**

- If YES, progress to LEVEL 2 at next session.
- If NO, repeat this exercise at next session.

**Stability Limits / Verticality Exercises**

**Forward Reaching for Object**

**Level 2:** Forward Reaching for Object Without UE Support With Narrow Base of Support

**Equipment:** Table, cup

**Instructions:** I am going to ask you to reach for the cup and hand it to me while maintaining your balance. Stand with your feet together. Reach for the object while trying to not to let your heels rise from the ground. Once you grab the cup, hand it to me. We will repeat this reaching exercise 10 times.

****Object should be set a distance that requires the participant to reach as far as possible without allowing heels to come off the surface.****

**Verbal Cues for Feedback:**

- Keep your feet together and bend at your hips while reaching.
- Allow your weight to shift toward the front of your feet as you reach.
- Keep your heels on the ground throughout the task.

**Successful Repetition:**

- Completes 10 reaches successfully without loss of balance (i.e. stepping strategy).
- Maintains heels on the ground for all 10 repetitions.

**Unsuccessful Repetition:**

- Unable to complete 10 repetitions without loss of balance (i.e. stepping strategy).
- Unable to maintain feet together AND/OR heels on the ground while reaching.

**Performance**

- **Set 1**
  - Successful Repetitions: ______ /10
  - Number of Verbal Cues Given: ______________
- **Set 2**
  - Successful Repetitions: ______ /10
  - Number of Verbal Cues Given: ______________
- **Set 3**
  - Successful Repetitions: ______ /10
  - Number of Verbal Cues Given: ______________

**Criteria for Progression**

- Successful performance of all repetitions and sets.
- No more than 2 verbal cues provided per set.

**Does the participant meet the criteria for progression? YES / NO**

- If YES, progress to LEVEL 3 at next session.
- If NO, repeat this exercise at next session.

**Stability Limits / Verticality Exercises**

**Forward Reaching for Object**

**Level 3:** Forward Reaching for Object Without UE Support With Narrow Base of Support With Eyes Closed.

**Equipment:** Table, cup

**Instructions:** I am going to ask you to reach for the cup and hand it to me while maintaining your balance. Stand with your feet together. Close your eyes. Reach for the object while trying to not to let your heels rise from the ground. Once you grab the cup, hand it to me. We will repeat this reaching exercise 10 times.

****Object should be set a distance that requires the participant to reach as far as possible without allowing heels to come off the surface.****

**Verbal Cues for Feedback:**

- Keep your feet together and bend at your hips while reaching.
- Allow your weight to shift toward the front of your feet as you reach.
- Keep your eyes closed and heels on the ground throughout the task.

**Successful Repetition:**

- Completes 10 reaches successfully without loss of balance (i.e. stepping strategy).
- Maintains heels on the ground for all 10 repetitions.
- Maintains eyes closed.

**Unsuccessful Repetition:**

- Unable to complete 10 repetitions without loss of balance (i.e. stepping strategy).
- Unable to maintain feet together AND/OR heels on the ground while reaching.
- Opens eyes.

**Performance**

- **Set 1**
  - Successful Repetitions: ______ /10
  - Number of Verbal Cues Given: ______________
- **Set 2**
  - Successful Repetitions: ______ /10
  - Number of Verbal Cues Given: ______________
- **Set 3**
  - Successful Repetitions: ______ /10
  - Number of Verbal Cues Given: ______________

**Criteria for Progression**

- Successful performance of all repetitions and sets.
- No more than 2 verbal cues provided per set.

**Does the participant meet the criteria for progression? YES / NO**

- If YES, progress to LEVEL 4 at next session.
- If NO, repeat this exercise at next session.

**Stability Limits / Verticality Exercises**

**Forward Reaching for Object**

**Level 4:** Forward Reaching for Object With Narrow Base of Support With Eyes Open on Foam.

**Equipment:** Table, cup, memory foam

**Instructions:** I am going to ask you to reach for the cup and hand it to me while maintaining your balance. Stand on this foam with your feet together. Reach for the object while trying to not to let your heels rise from the foam. Once you grab the cup, hand it to me. We will repeat this reaching exercise 10 times.

****Object should be set a distance that requires the participant to reach as far as possible without allowing heels to come off the surface.****

**Verbal Cues for Feedback:**

- Keep your feet together and bend at your hips while reaching.
- Allow your weight to shift toward the front of your feet as you reach.

**Successful Repetition:**

- Completes 10 reaches successfully without loss of balance (i.e. stepping strategy).
- Maintains heels on the foam for all 10 repetitions.

**Unsuccessful Repetition:**

- Unable to complete 10 repetitions without loss of balance (i.e. stepping strategy).
- Unable to maintain feet together AND/OR heels on the foam while reaching.

**Performance**

- **Set 1**
  - Successful Repetitions: ______ /10
  - Number of Verbal Cues Given: ______________
- **Set 2**
  - Successful Repetitions: ______ /10
  - Number of Verbal Cues Given: ______________
- **Set 3**
  - Successful Repetitions: ______ /10
  - Number of Verbal Cues Given: ______________

**Criteria for Progression**

- Successful performance of all repetitions and sets.
- No more than 2 verbal cues provided per set.

**Does the participant meet the criteria for progression? YES / NO**

- If YES, progress to LEVEL 5 at next session.
- If NO, repeat this exercise at next session.

**Stability Limits / Verticality Exercises**

**Forward Reaching for Object**

**Level 5:** Forward Reaching for Object With Narrow Base of Support With Eyes Closed on Foam.

**Equipment:** Table, cup, memory foam

**Instructions:** I am going to ask you to reach for the cup and hand it to me while maintaining your balance. Stand on this foam with your feet together. Close your eyes. Reach for the object while trying to not to let your heels rise from the foam. Once you grab the cup, hand it to me. We will repeat this reaching exercise 10 times.

****Object should be set a distance that requires the participant to reach as far as possible without allowing heels to come off the surface.****

**Verbal Cues for Feedback:**

- Keep your feet together and bend at your hips while reaching.
- Allow your weight to shift toward the front of your feet as you reach.
- Keep your eyes closed and heels on the foam.

**Successful Repetition:**

- Completes 10 reaches successfully without loss of balance (i.e. stepping strategy).
- Maintains heels on the foam for all 10 repetitions.
- Maintains eyes closed.

**Unsuccessful Repetition:**

- Unable to complete 10 repetitions without loss of balance (i.e. stepping strategy).
- Unable to maintain feet together AND/OR heels on the foam while reaching.
- Opens eyes.

**Performance**

- **Set 1**
  - Successful Repetitions: ______ /10
  - Number of Verbal Cues Given: ______________
- **Set 2**
  - Successful Repetitions: ______ /10
  - Number of Verbal Cues Given: ______________
- **Set 3**
  - Successful Repetitions: ______ /10
  - Number of Verbal Cues Given: ______________

**Criteria for Progression**

- Successful performance of all repetitions and sets.
- No more than 2 verbal cues provided per set.

**Does the participant meet the criteria for progression? YES / NO**

- If YES, progress to LEVEL 6 at next session.
- If NO, repeat this exercise at next session.

**Stability Limits / Verticality Exercises**

**Forward Reaching for Object**

**Level 6:** Forward Reaching for Object With Narrow Base of Support With Eyes Open on Theraband Stability Trainer Pads (Advanced Level Black Inflatable Pads).

**Equipment:** Table, cup, Theraband Stability Trainer Pads (Advanced Level Black Inflatable Pads)

**Instructions:** I am going to ask you to reach for the cup and hand it to me while maintaining your balance. Stand on these discs with your feet together. Reach for the object while trying to not to let your heels rise from the foam. Once you grab the cup, hand it to me. We will repeat this reaching exercise 10 times.

****Object should be set a distance that requires the participant to reach as far as possible without allowing heels to come off the surface.****

**Verbal Cues for Feedback:**

- Keep your feet together and bend at your hips while reaching.
- Allow your weight to shift toward the front of your feet as you reach.

**Successful Repetition:**

- Completes 10 reaches successfully without loss of balance (i.e. stepping strategy).
- Maintains heels on the Theraband Stability Trainer Pads (Advanced Level Black Inflatable Pads) for all 10 repetitions.

**Unsuccessful Repetition:**

- Unable to complete 10 repetitions without loss of balance (i.e. stepping strategy).
- Unable to maintain feet together AND/OR heels on the Theraband Stability Trainer Pads (Advanced Level Black Inflatable Pads) while reaching.

**Performance**

- **Set 1**
  - Successful Repetitions: ______ /10
  - Number of Verbal Cues Given: ______________
- **Set 2**
  - Successful Repetitions: ______ /10
  - Number of Verbal Cues Given: ______________
- **Set 3**
  - Successful Repetitions: ______ /10
  - Number of Verbal Cues Given: ______________

**Criteria for Progression**

- Successful performance of all repetitions and sets.
- No more than 2 verbal cues provided per set.

**Does the participant meet the criteria for progression? YES / NO**

- If YES, progress to LEVEL 7 at next session.
- If NO, repeat this exercise at next session.

**Stability Limits / Verticality Exercises**

**Forward Reaching for Object**

**Level 7:** Forward Reaching for Object With Narrow Base of Support With Eyes Closed on Theraband Stability Trainer Pads (Advanced Level Black Inflatable Pads).

**Equipment:** Table, cup, Theraband Stability Trainer Pads (Advanced Level Black Inflatable Pads)

**Instructions:** I am going to ask you to reach for the cup and hand it to me while maintaining your balance. Stand on these discs with your feet together. Close your eyes. Reach for the object while trying to not to let your heels rise from the discs. Once you grab the cup, hand it to me. We will repeat this reaching exercise 10 times.

****Object should be set a distance that requires the participant to reach as far as possible without allowing heels to come off the surface.****

**Verbal Cues for Feedback:**

- Keep your feet together and bend at your hips while reaching.
- Allow your weight to shift toward the front of your feet as you reach.
- Keep your eyes closed and heels on the discs.

**Successful Repetition:**

- Completes 10 reaches successfully without loss of balance (i.e. stepping strategy).
- Maintains heels on the discs for all 10 repetitions.
- Maintains eyes closed.

**Unsuccessful Repetition:**

- Unable to complete 10 repetitions without loss of balance (i.e. stepping strategy).
- Unable to maintain feet together AND/OR heels on the discs while reaching.
- Opens eyes.

**Performance**

- **Set 1**
  - Successful Repetitions: ______ /10
  - Number of Verbal Cues Given: ______________
- **Set 2**
  - Successful Repetitions: ______ /10
  - Number of Verbal Cues Given: ______________
- **Set 3**
  - Successful Repetitions: ______ /10
  - Number of Verbal Cues Given: ______________

**Criteria for Progression**

- Successful performance of all repetitions and sets.
- No more than 2 verbal cues provided per set.

**Does the participant meet the criteria for progression? YES / NO**

- If YES, progress to LEVEL 8 at next session.
- If NO, repeat this exercise at next session.

**Stability Limits / Verticality Exercises**

**Forward Reaching for Object**

**Level 8:** Forward Reaching for Object With Normal Base of Support With Eyes Open on BOSU ball.

**Equipment:** Table, cup, BOSU

**Instructions:** I am going to ask you to reach for the cup and hand it to me while maintaining your balance. Stand on this BOSU ball with your feet shoulder-width apart. Reach for the object while trying to not to let your heels rise from the surface. Once you grab the cup, hand it to me. We will repeat this reaching exercise 10 times.

****Object should be set a distance that requires the participant to reach as far as possible without allowing heels to come off the surface.****

**Verbal Cues for Feedback:**

- Keep your feet together and bend at your hips while reaching.
- Allow your weight to shift toward the front of your feet as you reach.

**Successful Repetition:**

- Completes 10 reaches successfully without loss of balance (i.e. stepping strategy).
- Maintains heels on the surface for all 10 repetitions.

**Unsuccessful Repetition:**

- Unable to complete 10 repetitions without loss of balance (i.e. stepping strategy).
- Unable to maintain feet together AND/OR heels on the surface while reaching.

**Performance**

- **Set 1**
  - Successful Repetitions: ______ /10
  - Number of Verbal Cues Given: ______________
- **Set 2**
  - Successful Repetitions: ______ /10
  - Number of Verbal Cues Given: ______________
- **Set 3**
  - Successful Repetitions: ______ /10
  - Number of Verbal Cues Given: ______________

**Criteria for Progression**

- Successful performance of all repetitions and sets.
- No more than 2 verbal cues provided per set.

**Does the participant meet the criteria for progression? YES / NO**

- If YES, continue this exercise for the remainder of your PT sessions.
- If NO, repeat this exercise at next session.

**Stability Limits / Verticality Exercises**

**Lateral Reaching for Object**

**Level 1:** Lateral Reaching (Right and Left) for Object Without UE Support

**Equipment:** Table, cup

Instructions: I am going to ask you to reach to the side for the cup and hand it to me while maintaining your balance. Stand with your feet shoulder-width apart. Reach to your side for the object while trying to not to step. Once you grab the cup, hand it to me. We will repeat this reaching exercise 10 times to each side.

****Object should be set a distance that requires the participant to reach as far as possible without allowing a step.****

**Verbal Cues for Feedback:**

- Bend at your knees and hips while reaching.
- Allow your weight to shift toward the side of your foot as you reach.
- Keep your feet on the ground throughout the task.

**Successful Repetition:**

- Completes 10 reaches successfully to each side without loss of balance (i.e. stepping strategy).
- Maintains feet on the ground for all 10 repetitions.

**Unsuccessful Repetition:**

- Unable to complete 10 repetitions without loss of balance (i.e. stepping strategy).

**Performance:**

- **Set 1**
  - **Successful Repetitions: R:____/10____ L:____/10____**
  - **Number of Verbal Cues: R:__________ L:__________**
- **Set 2**
  - **Successful Repetitions: R:____/10____ L:____/10____**
  - **Number of Verbal Cues: R:__________ L:__________**
- **Set 3**
  - **Successful Repetitions: R:____/10____ L:____/10____**
  - **Number of Verbal Cues: R:__________ L:__________**

**Criteria for Progression**

- Successful performance of all repetitions and sets.
- No more than 2 verbal cues provided per set.

**Does the participant meet the criteria for progression? YES / NO**

- If YES, progress to LEVEL 2 at next session.
- If NO, repeat this exercise at next session.

**Stability Limits / Verticality Exercises**

**Lateral Reaching for Object**

**Level 2:** Lateral Reaching for Object Without UE Support With Narrow Base of Support

**Equipment:** Table, cup

**Instructions:** I am going to ask you to reach to your side for the cup and hand it to me while maintaining your balance. Stand with your feet together. Reach for the object while trying to not to take a step. Once you grab the cup, hand it to me. We will repeat this reaching exercise 10 times to each side.

****Object should be set a distance that requires the participant to reach as far as possible without stepping.****

**Verbal Cues for Feedback:**

- Keep your feet together and bend at your hips and knees while reaching.
- Allow your weight to shift toward the side of your foot as you reach.
- Keep your feet on the ground throughout the task.

**Successful Repetition:**

- Completes 10 reaches successfully to each side without loss of balance (i.e. stepping strategy).
- Maintains feet on the ground for all 10 repetitions.

**Unsuccessful Repetition:**

- Unable to complete 10 repetitions without loss of balance (i.e. stepping strategy).
- Unable to maintain feet together AND/OR steps to regain balance.

**Performance**

- **Set 1**
  - **Successful Repetitions: R:____/10____ L:____/10____**
  - **Number of Verbal Cues: R:__________ L:__________**
- **Set 2**
  - **Successful Repetitions: R:____/10____ L:____/10____**
  - **Number of Verbal Cues: R:__________ L:__________**
- **Set 3**
  - **Successful Repetitions: R:____/10____ L:____/10____**
  - **Number of Verbal Cues: R:__________ L:__________**

**Criteria for Progression**

- Successful performance of all repetitions and sets.
- No more than 2 verbal cues provided per set.

**Does the participant meet the criteria for progression? YES / NO**

- If YES, progress to LEVEL 3 at next session.
- If NO, repeat this exercise at next session.

**Stability Limits / Verticality Exercises**

**Lateral Reaching for Object**

**Level 3:** Lateral Reaching for Object Without UE Support With Narrow Base of Support With Eyes Closed.

**Equipment:** Table, cup

**Instructions:** I am going to ask you to reach to your side for the cup and hand it to me while keeping your eyes closed and maintaining your balance. Stand with your feet together. Close your eyes. Reach to your side for the object while trying to not to step. Once you grab the cup, hand it to me. We will repeat this reaching exercise 10 times to each side.

****Object should be set a distance that requires the participant to reach as far as possible without stepping.****

**Verbal Cues for Feedback:**

- Keep your feet together and bend at your hips and knees while reaching.
- Allow your weight to shift toward the side of your feet as you reach.
- Keep your eyes closed and feet on the ground throughout the task.

**Successful Repetition:**

- Completes 10 reaches successfully without loss of balance (i.e. stepping strategy).
- Maintains feet on the ground for all 10 repetitions.
- Maintains eyes closed.

**Unsuccessful Repetition:**

- Unable to complete 10 repetitions without loss of balance (i.e. stepping strategy).
- Unable to maintain feet together AND/OR feet on the ground while reaching.
- Opens eyes.

**Performance**

- **Set 1**
  - **Successful Repetitions: R:____/10____ L:____/10____**
  - **Number of Verbal Cues: R:__________ L:__________**
- **Set 2**
  - **Successful Repetitions: R:____/10____ L:____/10____**
  - **Number of Verbal Cues: R:__________ L:__________**
- **Set 3**
  - **Successful Repetitions: R:____/10____ L:____/10____**
  - **Number of Verbal Cues: R:__________ L:__________**

**Criteria for Progression**

- Successful performance of all repetitions and sets.
- No more than 2 verbal cues provided per set.

**Does the participant meet the criteria for progression? YES / NO**

- If YES, progress to LEVEL 4 at next session.
- If NO, repeat this exercise at next session.

**Stability Limits / Verticality Exercises**

**Lateral Reaching for Object**

**Level 4:** Lateral Reaching for Object With Narrow Base of Support With Eyes Open on Foam.

**Equipment:** Table, cup, memory foam

**Instructions:** I am going to ask you to reach to your side for the cup and hand it to me while maintaining your balance. You will be standing on foam with your eyes open. Stand on this foam with your feet together. Reach to your side for the object while trying to not to let your heels rise from the foam. Once you grab the cup, hand it to me. We will repeat this reaching exercise 10 times to each side.

****Object should be set a distance that requires the participant to reach as far as possible without stepping.****

**Verbal Cues for Feedback:**

- Keep your feet together and bend at your hips and knees while reaching.
- Allow your weight to shift toward the side of your feet as you reach.

**Successful Repetition:**

- Completes 10 reaches successfully without loss of balance (i.e. stepping strategy).
- Maintains feet on the foam for all 10 repetitions.

**Unsuccessful Repetition:**

- Unable to complete 10 repetitions without loss of balance (i.e. stepping strategy).
- Unable to maintain feet together AND/OR feet on the foam while reaching.

**Performance**

- **Set 1**
  - **Successful Repetitions: R:____/10____ L:____/10____**
  - **Number of Verbal Cues: R:__________ L:__________**
- **Set 2**
  - **Successful Repetitions: R:____/10____ L:____/10____**
  - **Number of Verbal Cues: R:__________ L:__________**
- **Set 3**
  - **Successful Repetitions: R:____/10____ L:____/10____**
  - **Number of Verbal Cues: R:__________ L:__________**

**Criteria for Progression**

- Successful performance of all repetitions and sets.
- No more than 2 verbal cues provided per set.

**Does the participant meet the criteria for progression? YES / NO**

- If YES, progress to LEVEL 5 at next session.
- If NO, repeat this exercise at next session.

**Stability Limits / Verticality Exercises**

**Lateral Reaching for Object**

**Level 5:** Lateral Reaching for Object With Narrow Base of Support With Eyes Closed on Foam.

**Equipment:** Table, cup, memory foam

**Instructions:** I am going to ask you to reach to the side for the cup and hand it to me while maintaining your balance. You will be standing on foam with your eyes closed. Stand on this foam with your feet together. Close your eyes. Reach to your side for the object while trying to not to step. Once you grab the cup, hand it to me. We will repeat this reaching exercise 10 times to each side.

****Object should be set a distance that requires the participant to reach as far as possible without stepping.****

**Verbal Cues for Feedback:**

- Keep your feet together and bend at your hips and knees while reaching.
- Allow your weight to shift toward the side of your feet as you reach.
- Keep your eyes closed and feet on the foam.

**Successful Repetition:**

- Completes 10 reaches successfully without loss of balance (i.e. stepping strategy).
- Maintains feet on the foam for all 10 repetitions.
- Maintains eyes closed.

**Unsuccessful Repetition:**

- Unable to complete 10 repetitions without loss of balance (i.e. stepping strategy).
- Unable to maintain feet together AND/OR feet on the foam while reaching.
- Opens eyes.

**Performance**

- **Set 1**
  - **Successful Repetitions: R:____/10____ L:____/10____**
  - **Number of Verbal Cues: R:__________ L:__________**
- **Set 2**
  - **Successful Repetitions: R:____/10____ L:____/10____**
  - **Number of Verbal Cues: R:__________ L:__________**
- **Set 3**
  - **Successful Repetitions: R:____/10____ L:____/10____**
  - **Number of Verbal Cues: R:__________ L:__________**

**Criteria for Progression**

- Successful performance of all repetitions and sets.
- No more than 2 verbal cues provided per set.

**Does the participant meet the criteria for progression? YES / NO**

- If YES, progress to LEVEL 6 at next session.
- If NO, repeat this exercise at next session.

**Stability Limits / Verticality Exercises**

**Lateral Reaching for Object**

**Level 6:** Forward Reaching for Object With Narrow Base of Support With Eyes Open on Theraband Stability Trainer Pads (Advanced Level Black Inflatable Pads).

**Equipment:** Table, cup, Theraband Stability Trainer Pads (Advanced Level Black Inflatable Pads)

**Instructions:** I am going to ask you to reach for the cup and hand it to me while maintaining your balance. You will be standing on Theraband Stability Trainer Pads (Advanced Level Black Inflatable Pads) with your eyes open. Stand on these discs with your feet together. Reach to your side for the object while trying to not to step. Once you grab the cup, hand it to me. We will repeat this reaching exercise 10 times to each side.

****Object should be set a distance that requires the participant to reach as far as possible without stepping.****

**Verbal Cues for Feedback:**

- Keep your feet together and bend at your hips and knees while reaching.
- Allow your weight to shift toward the front of your feet as you reach.

**Successful Repetition:**

- Completes 10 reaches successfully without loss of balance (i.e. stepping strategy).
- Maintains feet on the Theraband Stability Trainer Pads (Advanced Level Black Inflatable Pads) for all 10 repetitions.

**Unsuccessful Repetition:**

- Unable to complete 10 repetitions without loss of balance (i.e. stepping strategy).
- Unable to maintain feet together AND/OR feet on the Theraband Stability Trainer Pads (Advanced Level Black Inflatable Pads) while reaching.

**Performance**

- **Set 1**
  - **Successful Repetitions: R:____/10____ L:____/10____**
  - **Number of Verbal Cues: R:__________ L:__________**
- **Set 2**
  - **Successful Repetitions: R:____/10____ L:____/10____**
  - **Number of Verbal Cues: R:__________ L:__________**
- **Set 3**
  - **Successful Repetitions: R:____/10____ L:____/10____**
  - **Number of Verbal Cues: R:__________ L:__________**

**Criteria for Progression**

- Successful performance of all repetitions and sets.
- No more than 2 verbal cues provided per set.

**Does the participant meet the criteria for progression? YES / NO**

- If YES, progress to LEVEL 7 at next session.
- If NO, repeat this exercise at next session.

**Stability Limits / Verticality Exercises**

**Lateral Reaching for Object**

**Level 7:** Lateral Reaching for Object With Narrow Base of Support With Eyes Closed on Theraband Stability Trainer Pads (Advanced Level Black Inflatable Pads).

**Equipment:** Table, cup, Theraband Stability Trainer Pads (Advanced Level Black Inflatable Pads)

**Instructions:** I am going to ask you to reach for the cup and hand it to me while maintaining your balance. You will be standing on Theraband Stability Trainer Pads (Advanced Level Black Inflatable Pads) with your eyes closed. Stand on these discs with your feet together. Close your eyes. Reach to your side for the object while trying to not to step. Once you grab the cup, hand it to me. We will repeat this reaching exercise 10 times to each side. Keep your eyes closed until you hand me the cup.

****Object should be set a distance that requires the participant to reach as far as possible without stepping.****

**Verbal Cues for Feedback:**

- Keep your feet together and bend at your hips and knees while reaching.
- Allow your weight to shift toward the side of your feet as you reach.
- Keep your eyes closed and feet on the discs.

**Successful Repetition:**

- Completes 10 reaches successfully without loss of balance (i.e. stepping strategy).
- Maintains feet on the discs for all 10 repetitions.
- Maintains eyes closed.

**Unsuccessful Repetition:**

- Unable to complete 10 repetitions without loss of balance (i.e. stepping strategy).
- Unable to maintain feet together AND/OR feet on the discs while reaching.
- Opens eyes.

**Performance**

- **Set 1**
  - **Successful Repetitions: R:____/10____ L:____/10____**
  - **Number of Verbal Cues: R:__________ L:__________**
- **Set 2**
  - **Successful Repetitions: R:____/10____ L:____/10____**
  - **Number of Verbal Cues: R:__________ L:__________**
- **Set 3**
  - **Successful Repetitions: R:____/10____ L:____/10____**
  - **Number of Verbal Cues: R:__________ L:__________**

**Criteria for Progression**

- Successful performance of all repetitions and sets.
- No more than 2 verbal cues provided per set.

**Does the participant meet the criteria for progression? YES / NO**

- If YES, progress to LEVEL 8 at next session.
- If NO, repeat this exercise at next session.

**Stability Limits / Verticality Exercises**

**Lateral Reaching for Object**

**Level 8:** Lateral Reaching for Object With Normal Base of Support With Eyes Open on BOSU ball.

**Equipment:** Table, cup, BOSU

**Instructions:** I am going to ask you to reach to your side for the cup and hand it to me while maintaining your balance. Stand on this BOSU ball with your feet shoulder-width apart. Reach for the object while trying to not to step from the surface. Once you grab the cup, hand it to me. We will repeat this reaching exercise 10 times to each side.

****Object should be set a distance that requires the participant to reach as far as possible without stepping.****

**Verbal Cues for Feedback:**

- Keep your feet together and bend at your hips and knees while reaching.
- Allow your weight to shift toward the side of your feet as you reach.

**Successful Repetition:**

- Completes 10 reaches successfully without loss of balance (i.e. stepping strategy).
- Maintains feet on the surface for all 10 repetitions.

**Unsuccessful Repetition:**

- Unable to complete 10 repetitions without loss of balance (i.e. stepping strategy).
- Unable to maintain feet together AND/OR feet on the surface while reaching.

**Performance**

- **Set 1**
  - **Successful Repetitions: R:____/10____ L:____/10____**
  - **Number of Verbal Cues: R:__________ L:__________**
- **Set 2**
  - **Successful Repetitions: R:____/10____ L:____/10____**
  - **Number of Verbal Cues: R:__________ L:__________**
- **Set 3**
  - **Successful Repetitions: R:____/10____ L:____/10____**
  - **Number of Verbal Cues: R:__________ L:__________**

**Criteria for Progression**

- Successful performance of all repetitions and sets.
- No more than 2 verbal cues provided per set.

**Does the participant meet the criteria for progression? YES / NO**

- If YES, continue this exercise for the remainder of your PT sessions.
- If NO, repeat this exercise at next session.

Anticipatory Postural Adjustments – Exercises

**Multi-Directional Stepping**

**Level 1: Multi-Directional Stepping (FWD, BCK, LAT) WITH Upper Extremity Assistance**

**Equipment: Treadmill rail, chair, or wall.**

**Instructions:** I will ask you step in 4 different directions (Forward, Backward, Left/Right). Start with feet even, shoulder-width apart. Please one hand on the support surface, shift your weight to the stance leg, and step in the specified direction. Step to a distance that is comfortable for you and then return to the starting position. Alternate this movement for a total of 10 steps with each leg. Try to minimize the amount of body sway while tapping the step and minimize the amount of pressure you put through your hands on the support surface.

**Verbal Cues for Feedback:**

- Remember to shift your weight so that your center of mass is over the stance foot.
- Remember to minimize the pressure you put through your hands on the support surface.
- Try to minimize the amount that you sway.

**Successful Repetition:**

- Completes 10 steps with each foot in specified direction, no more than ankle strategy to maintain balance.
- Maintains minimal pressure on support surface.

**Unsuccessful Repetition:**

- Unable to complete 10 steps with each foot AND/OR uses hip strategy or more to maintain balance.
- Maintains more than minimal pressure on support surface.

**Performance:**

- **Set 1**
  - **Successful Repetitions: FWD:____/20____ BCK:____/20____ LAT:____/20___**
  - **Number of Verbal Cues: FWD:________ BCK:________ LAT:_______**
- **Set 2**
  - **Successful Repetitions: FWD:____/20____ BCK:____/20____ LAT:____/20___**
  - **Number of Verbal Cues: FWD:________ BCK:________ LAT:_______**
- **Set 3**
  - **Successful Repetitions: FWD:____/20____ BCK:____/20____ LAT:____/20___**
  - **Number of Verbal Cues: FWD:________ BCK:________ LAT:_______**

**Criteria for Progression:**

- **Successful performance of all repetitions and sets.**
- **No more than 2 verbal cues provided per set per side.**

**Does the participant meet the criteria for progression? YES / NO**

- **If YES, progress to LEVEL 2.**
- **If NO, repeat this exercise at next session.**

Anticipatory Postural Adjustments – Exercises

**Multi-Directional Stepping**

**Level 2: Multi-Directional Stepping (FWD, BCK, LAT) WITH Upper Extremity Assistance WITH Large Step**

**Equipment: Treadmill rail, chair, or wall.**

**Instructions:** I will ask you step in 4 different directions (Forward, Backward, Left/Right). Start with feet even, shoulder-width apart. Please one hand on the support surface, shift your weight to the stance leg, and step in the specified direction. Step as far as you can and then return to the starting position. Alternate this movement for a total of 10 steps with each leg. Try to minimize the amount of body sway while tapping the step and minimize the amount of pressure you put through your hands on the support surface.

**Verbal Cues for Feedback:**

- Remember to shift your weight so that your center of mass is over the stance foot.
- Remember to minimize the pressure you put through your hands on the support surface.
- Remember to take a large step.
- Try to minimize the amount that you sway.

**Successful Repetition:**

- Completes 10 steps with each foot in specified direction, no more than ankle strategy to maintain balance.
- Maintains minimal pressure on support surface.

**Unsuccessful Repetition:**

- Unable to complete 10 steps with each foot AND/OR uses hip strategy or more to maintain balance.
- Maintains more than minimal pressure on support surface.

**Performance:**

- **Set 1**
  - **Successful Repetitions: FWD:____/20____ BCK:____/20____ LAT:____/20___**
  - **Number of Verbal Cues: FWD:________ BCK:________ LAT:_______**
- **Set 2**
  - **Successful Repetitions: FWD:____/20____ BCK:____/20____ LAT:____/20___**
  - **Number of Verbal Cues: FWD:________ BCK:________ LAT:_______**
- **Set 3**
  - **Successful Repetitions: FWD:____/20____ BCK:____/20____ LAT:____/20___**
  - **Number of Verbal Cues: FWD:________ BCK:________ LAT:_______**

**Criteria for Progression:**

- **Successful performance of all repetitions and sets.**
- **No more than 2 verbal cues provided per set per side.**

**Does the participant meet the criteria for progression? YES / NO**

- **If YES, progress to LEVEL 3.**
- **If NO, repeat this exercise at next session.**

Anticipatory Postural Adjustments – Exercises

**Multi-Directional Stepping**

**Level 3: Multi-Directional Stepping (FWD, BCK, LAT) WITHOUT Upper Extremity Assistance**

**Equipment: Treadmill rail, chair, or wall.**

**Instructions:** I will ask you step in 4 different directions (Forward, Backward, Left/Right). Start with feet even, shoulder-width apart. Keep one hand just above the support surface. Shift your weight to the stance leg, and step in the specified direction. Step to a distance that is comfortable for you and then return to the starting position. Alternate this movement for a total of 10 steps with each leg. Try to minimize the amount of body sway while tapping the step and try not to grab onto the support surface.

**Verbal Cues for Feedback:**

- Remember to shift your weight so that your center of mass is over the stance foot.
- Try to minimize the amount that you sway.
- Try not to grab onto the support surface.

**Successful Repetition:**

- Completes 10 steps with each foot in specified direction, no more than ankle strategy to maintain balance.
- Does not grab support surface.

**Unsuccessful Repetition:**

- Unable to complete 10 steps with each foot AND/OR uses hip strategy or more to maintain balance.
- Grabs support surface.

**Performance:**

- **Set 1**
  - **Successful Repetitions: FWD:____/20____ BCK:____/20____ LAT:____/20___**
  - **Number of Verbal Cues: FWD:________ BCK:________ LAT:_______**
- **Set 2**
  - **Successful Repetitions: FWD:____/20____ BCK:____/20____ LAT:____/20___**
  - **Number of Verbal Cues: FWD:________ BCK:________ LAT:_______**
- **Set 3**
  - **Successful Repetitions: FWD:____/20____ BCK:____/20____ LAT:____/20___**
  - **Number of Verbal Cues: FWD:________ BCK:________ LAT:_______**

**Criteria for Progression:**

- **Successful performance of all repetitions and sets.**
- **No more than 2 verbal cues provided per set per side.**

**Does the participant meet the criteria for progression? YES / NO**

- **If YES, progress to LEVEL 4.**
- **If NO, repeat this exercise at next session.**

Anticipatory Postural Adjustments – Exercises

**Multi-Directional Stepping**

**Level 4: Multi-Directional Stepping (FWD, BCK, LAT) WITHOUT Upper Extremity Assistance WITH Large Step**

**Equipment: Treadmill rail, chair, or wall.**

**Instructions:** I will ask you step in 4 different directions (Forward, Backward, Left/Right). Start with feet even, shoulder-width apart. Keep one hand just above the support surface. Shift your weight to the stance leg, and step in the specified direction. Step as far as you can and then return to the starting position. Alternate this movement for a total of 10 steps with each leg. Try to minimize the amount of body sway while tapping the step and try not to grab onto the support surface.

**Verbal Cues for Feedback:**

- Remember to shift your weight so that your center of mass is over the stance foot.
- Try to minimize the amount that you sway.
- Remember to take a large step.
- Try not to grab onto the support surface.

**Successful Repetition:**

- Completes 10 steps with each foot in specified direction, no more than ankle strategy to maintain balance.
- Does not grab support surface.

**Unsuccessful Repetition:**

- Unable to complete 10 steps with each foot AND/OR uses hip strategy or more to maintain balance.
- Grabs support surface.

**Performance:**

- **Set 1**
  - **Successful Repetitions: FWD:____/20____ BCK:____/20____ LAT:____/20___**
  - **Number of Verbal Cues: FWD:________ BCK:________ LAT:_______**
- **Set 2**
  - **Successful Repetitions: FWD:____/20____ BCK:____/20____ LAT:____/20___**
  - **Number of Verbal Cues: FWD:________ BCK:________ LAT:_______**
- **Set 3**
  - **Successful Repetitions: FWD:____/20____ BCK:____/20____ LAT:____/20___**
  - **Number of Verbal Cues: FWD:________ BCK:________ LAT:_______**

**Criteria for Progression:**

- **Successful performance of all repetitions and sets.**
- **No more than 2 verbal cues provided per set per side.**

**Does the participant meet the criteria for progression? YES / NO**

- **If YES, progress to LEVEL 5.**
- **If NO, repeat this exercise at next session.**

Anticipatory Postural Adjustments – Exercises

**Multi-Directional Stepping**

**Level 5: Multi-Directional Stepping (FWD, BCK, LAT) WITHOUT Upper Extremity Assistance WITH EYES CLOSED**

**Equipment: Treadmill rail, chair, or wall.**

**Instructions:** I will ask you step in 4 different directions (Forward, Backward, Left/Right). Start with feet even, shoulder-width apart. Keep one hand just above the support surface. Close your eyes, shift your weight to the stance leg, and step in the specified direction. Step to a distance that is comfortable for you and then return to the starting position. Alternate this movement for a total of 10 steps with each leg. Keep your eyes closed throughout the task. Try to minimize the amount of body sway while tapping the step and try not to grab onto the support surface.

**Verbal Cues for Feedback:**

- Remember to shift your weight so that your center of mass is over the stance foot.
- Try to minimize the amount that you sway.
- Keep your eyes closed and try not to grab onto the support surface.

**Successful Repetition:**

- Completes 10 steps with each foot in specified direction, no more than ankle strategy to maintain balance.
- Does not grab support surface.
- Maintains eyes closed.

**Unsuccessful Repetition:**

- Unable to complete 10 steps with each foot AND/OR uses hip strategy or more to maintain balance.
- Grabs support surface.
- Opens eyes.

**Performance:**

- **Set 1**
  - **Successful Repetitions: FWD:____/20____ BCK:____/20____ LAT:____/20___**
  - **Number of Verbal Cues: FWD:________ BCK:________ LAT:_______**
- **Set 2**
  - **Successful Repetitions: FWD:____/20____ BCK:____/20____ LAT:____/20___**
  - **Number of Verbal Cues: FWD:________ BCK:________ LAT:_______**
- **Set 3**
  - **Successful Repetitions: FWD:____/20____ BCK:____/20____ LAT:____/20___**
  - **Number of Verbal Cues: FWD:________ BCK:________ LAT:_______**

**Criteria for Progression:**

- **Successful performance of all repetitions and sets.**
- **No more than 2 verbal cues provided per set per side.**

**Does the participant meet the criteria for progression? YES / NO**

- **If YES, progress to LEVEL 6.**
- **If NO, repeat this exercise at next session.**

Anticipatory Postural Adjustments – Exercises

**Multi-Directional Stepping**

**Level 6: Multi-Directional Stepping (FWD, BCK, LAT) WITHOUT UE Assistance WITH Large Step WITH EYES CLOSED**

**Equipment: Treadmill rail, chair, or wall.**

**Instructions:** I will ask you step in 4 different directions (Forward, Backward, Left/Right). Start with feet even, shoulder-width apart. Keep one hand just above the support surface. Close your eyes, shift your weight to the stance leg, and step in the specified direction. Step as far as you can and then return to the starting position. Alternate this movement for a total of 10 steps with each leg. Keep your eyes closed throughout the task. Try to minimize the amount of body sway while tapping the step and try not to grab onto the support surface.

**Verbal Cues for Feedback:**

- Remember to shift your weight so that your center of mass is over the stance foot.
- Remember to take a large step.
- Try to minimize the amount that you sway.
- Keep your eyes closed and try not to grab onto the support surface.

**Successful Repetition:**

- Completes 10 steps with each foot in specified direction, no more than ankle strategy to maintain balance.
- Does not grab support surface.
- Maintains eyes closed.

**Unsuccessful Repetition:**

- Unable to complete 10 steps with each foot AND/OR uses hip strategy or more to maintain balance.
- Grabs support surface.
- Opens eyes.

**Performance:**

- **Set 1**
  - **Successful Repetitions: FWD:____/20____ BCK:____/20____ LAT:____/20___**
  - **Number of Verbal Cues: FWD:________ BCK:________ LAT:_______**
- **Set 2**
  - **Successful Repetitions: FWD:____/20____ BCK:____/20____ LAT:____/20___**
  - **Number of Verbal Cues: FWD:________ BCK:________ LAT:_______**
- **Set 3**
  - **Successful Repetitions: FWD:____/20____ BCK:____/20____ LAT:____/20___**
  - **Number of Verbal Cues: FWD:________ BCK:________ LAT:_______**

**Criteria for Progression:**

- **Successful performance of all repetitions and sets.**
- **No more than 2 verbal cues provided per set per side.**

**Does the participant meet the criteria for progression? YES / NO**

- **If YES, progress to LEVEL 6.**
- **If NO, repeat this exercise at next session.**

Anticipatory Postural Adjustments – Exercises

**Multi-Directional Stepping**

**Level 6: Multi-Directional Stepping (FWD, BCK, LAT) WITHOUT Upper Extremity Assistance WITH DUAL TASK**

**Equipment: Treadmill rail, chair, or wall AND cup of water.**

**Instructions:** I will ask you step in 4 different directions (Forward, Backward, Left/Right). Start with feet even, shoulder-width apart. Keep one hand just above the support surface and hold this cup of water in the other. Shift your weight to the stance leg, and step in the specified direction. Step to a distance that is comfortable for you and then return to the starting position. Try not to spill the water. Alternate this movement for a total of 10 steps with each leg. Try to minimize the amount of body sway while tapping the step and try not to grab onto the support surface.

**Verbal Cues for Feedback:**

- Remember to shift your weight so that your center of mass is over the stance foot.
- Try to minimize the amount that you sway.
- Try not to grab onto the support surface.
- Try not to spill the water.

**Successful Repetition:**

- Completes 10 steps with each foot in specified direction, no more than ankle strategy to maintain balance.
- Does not grab support surface.
- No water spills.

**Unsuccessful Repetition:**

- Unable to complete 10 steps with each foot AND/OR uses hip strategy or more to maintain balance.
- Grabs support surface.
- Water spills.

**Performance:**

- **Set 1**
  - **Successful Repetitions: FWD:____/20____ BCK:____/20____ LAT:____/20___**
  - **Number of Verbal Cues: FWD:________ BCK:________ LAT:_______**
- **Set 2**
  - **Successful Repetitions: FWD:____/20____ BCK:____/20____ LAT:____/20___**
  - **Number of Verbal Cues: FWD:________ BCK:________ LAT:_______**
- **Set 3**
  - **Successful Repetitions: FWD:____/20____ BCK:____/20____ LAT:____/20___**
  - **Number of Verbal Cues: FWD:________ BCK:________ LAT:_______**

**Criteria for Progression:**

- **Successful performance of all repetitions and sets.**
- **No more than 2 verbal cues provided per set per side.**

**Does the participant meet the criteria for progression? YES / NO**

- **If YES, progress to LEVEL 7.**
- **If NO, repeat this exercise at next session.**

Anticipatory Postural Adjustments – Exercises

**Multi-Directional Stepping**

**Level 7: Multi-Directional Stepping (FWD, BCK, LAT) WITHOUT UE Assistance WITH LARGE STEP WITH DUAL TASK**

**Equipment: Treadmill rail, chair, or wall AND cup of water.**

**Instructions:** I will ask you step in 4 different directions (Forward, Backward, Left/Right). Start with feet even, shoulder-width apart. Keep one hand just above the support surface and hold this cup of water in the other. Shift your weight to the stance leg, and step in the specified direction. Step as far as you can and then return to the starting position. Try not to spill the water. Alternate this movement for a total of 10 steps with each leg. Try to minimize the amount of body sway while tapping the step and try not to grab onto the support surface.

**Verbal Cues for Feedback:**

- Remember to shift your weight so that your center of mass is over the stance foot.
- Remember to take a large step.
- Try to minimize the amount that you sway and try not to grab onto the support surface.
- Try not to spill the water.

**Successful Repetition:**

- Completes 10 steps with each foot in specified direction, no more than ankle strategy to maintain balance.
- Does not grab support surface.
- No water spills.

**Unsuccessful Repetition:**

- Unable to complete 10 steps with each foot AND/OR uses hip strategy or more to maintain balance.
- Grabs support surface.
- Water spills.

**Performance:**

- **Set 1**
  - **Successful Repetitions: FWD:____/20____ BCK:____/20____ LAT:____/20___**
  - **Number of Verbal Cues: FWD:________ BCK:________ LAT:_______**
- **Set 2**
  - **Successful Repetitions: FWD:____/20____ BCK:____/20____ LAT:____/20___**
  - **Number of Verbal Cues: FWD:________ BCK:________ LAT:_______**
- **Set 3**
  - **Successful Repetitions: FWD:____/20____ BCK:____/20____ LAT:____/20___**
  - **Number of Verbal Cues: FWD:________ BCK:________ LAT:_______**

**Criteria for Progression:**

- **Successful performance of all repetitions and sets.**
- **No more than 2 verbal cues provided per set per side.**

**Does the participant meet the criteria for progression? YES / NO**

- **If YES, progress to LEVEL 8.**
- **If NO, repeat this exercise at next session.**

Anticipatory Postural Adjustments – Exercises

**Multi-Directional Stepping**

**Level 8: Multi-Directional Stepping (FWD, BCK, LAT) W/O UE Assistance W/LARGE STEP, W/DUAL TASK EYES CLOSED**

**Equipment: Treadmill rail, chair, or wall AND cup of water.**

**Instructions:** I will ask you step in 4 different directions (Forward, Backward, Left/Right). Start with feet even, shoulder-width apart. Keep one hand just above the support surface and hold this cup of water in the other. Close your eyes, shift your weight to the stance leg, and step in the specified direction. Step as far as you can and then return to the starting position. Keep your eyes closed throughout the task while trying not to spill the water. Alternate this movement for a total of 10 steps with each leg. Try to minimize the amount of body sway while tapping the step and try not to grab onto the support surface.

**Verbal Cues for Feedback:**

- Remember to shift your weight so that your center of mass is over the stance foot.
- Remember to keep your eyes closed and take a large step.
- Try to minimize the amount that you sway and try not to grab onto the support surface.
- Try not to spill the water.

**Successful Repetition:**

- Completes 10 steps with each foot in specified direction, no more than ankle strategy to maintain balance.
- Does not grab support surface.
- No water spills.

**Unsuccessful Repetition:**

- Unable to complete 10 steps with each foot AND/OR uses hip strategy or more to maintain balance.
- Grabs support surface.
- Water spills.

**Performance:**

- **Set 1**
  - **Successful Repetitions: FWD:____/20____ BCK:____/20____ LAT:____/20___**
  - **Number of Verbal Cues: FWD:________ BCK:________ LAT:_______**
- **Set 2**
  - **Successful Repetitions: FWD:____/20____ BCK:____/20____ LAT:____/20___**
  - **Number of Verbal Cues: FWD:________ BCK:________ LAT:_______**
- **Set 3**
  - **Successful Repetitions: FWD:____/20____ BCK:____/20____ LAT:____/20___**
  - **Number of Verbal Cues: FWD:________ BCK:________ LAT:_______**

**Criteria for Progression:**

- **Successful performance of all repetitions and sets.**
- **No more than 2 verbal cues provided per set per side.**

**Does the participant meet the criteria for progression? YES / NO**

- **If YES, continue this exercise for the remainder of the participant’s PT sessions.**
- **If NO, repeat this exercise at next session.**

Anticipatory Postural Adjustments – Exercises

**Standing Step Up and Over Obstacle**

**Level 1: Standing Step Up and Over Small Obstacle WITH Upper Extremity Assistance**

**Equipment: Treadmill rail, chair, or wall for support AND foam roll (3 in. diameter).**

**Instructions:** I will ask you step up and over this obstacle, tap your heel on the floor, and return to the starting position. Please place your hand on the support surface, shift your weight to the stance leg and step up and over the obstacle with the opposite leg and tap your heel on the floor. Then return to the starting position while avoiding touching the object. Perform 10 consecutive taps with the right leg. Then do the same task stepping with the left. Try to minimize the amount of body sway while tapping the step and minimize the amount of pressure you put through your hands on the support surface.

**Verbal Cues for Feedback:**

- Remember to shift your weight so that your center of mass is over the stance foot.
- Remember to minimize the pressure you put through your hands on the support surface.
- Try to minimize the amount that you sway.

**Successful Repetition:**

- Completes 10 step overs on each foot with no more than ankle strategy to maintain balance.
- Maintains minimal pressure on support surface.

**Unsuccessful Repetition:**

- Unable to complete 10 step overs on each foot AND/OR uses hip strategy or more to maintain balance.
- Maintains more than minimal pressure on support surface.

**Performance:**

- **Set 1**
  - **Successful Repetitions: R:____/10____ L:____/10____**
  - **Number of Verbal Cues: R:__________ L:__________**
- **Set 2**
  - **Successful Repetitions: R:____/10____ L:____/10____**
  - **Number of Verbal Cues: R:__________ L:__________**
- **Set 3**
  - **Successful Repetitions: R:____/10____ L:____/10____**
  - **Number of Verbal Cues: R:__________ L:__________**

**Criteria for Progression:**

- **Successful performance of all repetitions and sets.**
- **No more than 2 verbal cues provided per set per side.**

**Does the participant meet the criteria for progression? YES / NO**

- **If YES, progress to LEVEL 2.**
- **If NO, repeat this exercise at next session.**

Anticipatory Postural Adjustments – Exercises

**Standing Step Up and Over Obstacle**

**Level 2: Standing Step Up and Over Small Obstacle WITH Upper Extremity Assistance**

**Equipment: Treadmill rail, chair, or wall for support AND foam roll (6 in. diameter).**

**Instructions:** I will ask you step up and over this obstacle, tap your heel on the floor, and return to the starting position. Please place your hand on the support surface, shift your weight to the stance leg and step up and over the obstacle with the opposite leg and tap your heel on the floor. Then return to the starting position while avoiding touching the object. Perform 10 consecutive taps with the right leg. Then do the same task stepping with the left. Try to minimize the amount of body sway while tapping the step and minimize the amount of pressure you put through your hands on the support surface.

**Verbal Cues for Feedback:**

- Remember to shift your weight so that your center of mass is over the stance foot.
- Remember to minimize the pressure you put through your hands on the support surface.
- Try to minimize the amount that you sway.

**Successful Repetition:**

- Completes 10 step overs on each foot with no more than ankle strategy to maintain balance.
- Maintains minimal pressure on support surface.

**Unsuccessful Repetition:**

- Unable to complete 10 step overs on each foot AND/OR uses hip strategy or more to maintain balance.
- Maintains more than minimal pressure on support surface.

**Performance:**

- **Set 1**
  - **Successful Repetitions: R:____/10____ L:____/10____**
  - **Number of Verbal Cues: R:__________ L:__________**
- **Set 2**
  - **Successful Repetitions: R:____/10____ L:____/10____**
  - **Number of Verbal Cues: R:__________ L:__________**
- **Set 3**
  - **Successful Repetitions: R:____/10____ L:____/10____**
  - **Number of Verbal Cues: R:__________ L:__________**

**Criteria for Progression:**

- **Successful performance of all repetitions and sets.**
- **No more than 2 verbal cues provided per set per side.**

**Does the participant meet the criteria for progression? YES / NO**

- **If YES, progress to LEVEL 3.**
- **If NO, repeat this exercise at next session.**

Anticipatory Postural Adjustments – Exercises

**Standing Step Up and Over Obstacle**

**Level 3: Standing Step Up and Over Small Obstacle WITHOUT Upper Extremity Assistance**

**Equipment: Treadmill rail, chair, or wall for support AND foam roll (3 in. diameter).**

**Instructions:** I will ask you step up and over this obstacle, tap your heel on the floor, and return to the starting position. Shift your weight to the stance leg and step up and over the obstacle with the opposite leg and tap your heel on the floor. Then return to the starting position while avoiding touching the object. Perform 10 consecutive taps with the right leg. Then do the same task stepping with the left. Try to minimize the amount of body sway while tapping the step. I will be here to guard you for safety.

**Verbal Cues for Feedback:**

- Remember to shift your weight so that your center of mass is over the stance foot.
- Try to minimize the amount that you sway.

**Successful Repetition:**

- Completes 10 step overs on each foot with no more than ankle strategy to maintain balance.
- Does not require use of support surface or physical assistance to maintain balance.

**Unsuccessful Repetition:**

- Unable to complete 10 step overs on each foot AND/OR uses hip strategy or more to maintain balance.
- Requires use of support surface or physical assistance to maintain balance.

**Performance:**

- **Set 1**
  - **Successful Repetitions: R:____/10____ L:____/10____**
  - **Number of Verbal Cues: R:__________ L:__________**
- **Set 2**
  - **Successful Repetitions: R:____/10____ L:____/10____**
  - **Number of Verbal Cues: R:__________ L:__________**
- **Set 3**
  - **Successful Repetitions: R:____/10____ L:____/10____**
  - **Number of Verbal Cues: R:__________ L:__________**

**Criteria for Progression:**

- **Successful performance of all repetitions and sets.**
- **No more than 2 verbal cues provided per set per side.**

**Does the participant meet the criteria for progression? YES / NO**

- **If YES, progress to LEVEL 4.**
- **If NO, repeat this exercise at next session.**

Anticipatory Postural Adjustments – Exercises

**Standing Step Up and Over Obstacle**

**Level 4: Standing Step Up and Over Small Obstacle WITHOUT Upper Extremity Assistance**

**Equipment: Treadmill rail, chair, or wall for support AND foam roll (6 in. diameter).**

**Instructions:** I will ask you step up and over this obstacle, tap your heel on the floor, and return to the starting position. Shift your weight to the stance leg and step up and over the obstacle with the opposite leg and tap your heel on the floor. Then return to the starting position while avoiding touching the object. Perform 10 consecutive taps with the right leg. Then do the same task stepping with the left. Try to minimize the amount of body sway while tapping the step. I will be here to guard you for safety.

**Verbal Cues for Feedback:**

- Remember to shift your weight so that your center of mass is over the stance foot.
- Try to minimize the amount that you sway.

**Successful Repetition:**

- Completes 10 step overs on each foot with no more than ankle strategy to maintain balance.
- Does not require use of support surface or physical assistance to maintain balance.

**Unsuccessful Repetition:**

- Unable to complete 10 step overs on each foot AND/OR uses hip strategy or more to maintain balance.
- Requires use of support surface or physical assistance to maintain balance.

**Performance:**

- **Set 1**
  - **Successful Repetitions: R:____/10____ L:____/10____**
  - **Number of Verbal Cues: R:__________ L:__________**
- **Set 2**
  - **Successful Repetitions: R:____/10____ L:____/10____**
  - **Number of Verbal Cues: R:__________ L:__________**
- **Set 3**
  - **Successful Repetitions: R:____/10____ L:____/10____**
  - **Number of Verbal Cues: R:__________ L:__________**

**Criteria for Progression:**

- **Successful performance of all repetitions and sets.**
- **No more than 2 verbal cues provided per set per side.**

**Does the participant meet the criteria for progression? YES / NO**

- **If YES, progress to LEVEL 5.**
- **If NO, repeat this exercise at next session.**

Anticipatory Postural Adjustments – Exercises

**Standing Step Up and Over Obstacle**

**Level 5: Standing Step Up and Over Small Obstacle WITHOUT Upper Extremity Assistance While on FOAM**

**Equipment: Treadmill rail, chair, or wall for support, foam Pads, AND foam roll (3 in. diameter).**

**Instructions:** I will ask you step up and over this obstacle, tap your heel on the floor, and return to the starting position. You will do this while standing on a foam Pads. Shift your weight to the stance leg and step up and over the obstacle with the opposite leg and tap your heel on the floor. Then return to the starting position while avoiding touching the object. Perform 10 consecutive taps with the right leg. Then do the same task stepping with the left. Try to minimize the amount of body sway while tapping the step. I will be here to guard you for safety.

**Verbal Cues for Feedback:**

- Remember to shift your weight so that your center of mass is over the stance foot.
- Try to minimize the amount that you sway.

**Successful Repetition:**

- Completes 10 step overs on each foot with no more than ankle strategy to maintain balance.
- Does not require use of support surface or physical assistance to maintain balance.

**Unsuccessful Repetition:**

- Unable to complete 10 step overs on each foot AND/OR uses hip strategy or more to maintain balance.
- Requires use of support surface or physical assistance to maintain balance.

**Performance:**

- **Set 1**
  - **Successful Repetitions: R:____/10____ L:____/10____**
  - **Number of Verbal Cues: R:__________ L:__________**
- **Set 2**
  - **Successful Repetitions: R:____/10____ L:____/10____**
  - **Number of Verbal Cues: R:__________ L:__________**
- **Set 3**
  - **Successful Repetitions: R:____/10____ L:____/10____**
  - **Number of Verbal Cues: R:__________ L:__________**

**Criteria for Progression:**

- **Successful performance of all repetitions and sets.**
- **No more than 2 verbal cues provided per set per side.**

**Does the participant meet the criteria for progression? YES / NO**

- **If YES, progress to LEVEL 6.**
- **If NO, repeat this exercise at next session.**

Anticipatory Postural Adjustments – Exercises

**Standing Step Up and Over Obstacle**

**Level 6: Standing Step Up and Over Small Obstacle WITHOUT Upper Extremity Assistance While on FOAM**

**Equipment: Treadmill rail, chair, or wall for support, foam Pads, AND foam roll (6 in. diameter).**

**Instructions:** I will ask you step up and over this obstacle, tap your heel on the floor, and return to the starting position. You will do this while standing on a foam Pads. Shift your weight to the stance leg and step up and over the obstacle with the opposite leg and tap your heel on the floor. Then return to the starting position while avoiding touching the object. Perform 10 consecutive taps with the right leg. Then do the same task stepping with the left. Try to minimize the amount of body sway while tapping the step. I will be here to guard you for safety.

**Verbal Cues for Feedback:**

- Remember to shift your weight so that your center of mass is over the stance foot.
- Try to minimize the amount that you sway.

**Successful Repetition:**

- Completes 10 step overs on each foot with no more than ankle strategy to maintain balance.
- Does not require use of support surface or physical assistance to maintain balance.

**Unsuccessful Repetition:**

- Unable to complete 10 step overs on each foot AND/OR uses hip strategy or more to maintain balance.
- Requires use of support surface or physical assistance to maintain balance.

**Performance:**

- **Set 1**
  - **Successful Repetitions: R:____/10____ L:____/10____**
  - **Number of Verbal Cues: R:__________ L:__________**
- **Set 2**
  - **Successful Repetitions: R:____/10____ L:____/10____**
  - **Number of Verbal Cues: R:__________ L:__________**
- **Set 3**
  - **Successful Repetitions: R:____/10____ L:____/10____**
  - **Number of Verbal Cues: R:__________ L:__________**

**Criteria for Progression:**

- **Successful performance of all repetitions and sets.**
- **No more than 2 verbal cues provided per set per side.**

**Does the participant meet the criteria for progression? YES / NO**

- **If YES, progress to LEVEL 7.**
- **If NO, repeat this exercise at next session.**

Anticipatory Postural Adjustments – Exercises

**Standing Step Up and Over Obstacle**

**Level 7: Standing Step Up and Over Small Obstacle WITHOUT UE Assistance While on FOAM WITH DUAL TASK**

**Equipment: Treadmill rail, chair, or wall for support AND foam roll (3 in. diameter).**

**Instructions:** I will ask you step up and over this obstacle, tap your heel on the floor, and return to the starting position. You will complete this task while standing on foam and holding a glass of water. Shift your weight to the stance leg and step up and over the obstacle with the opposite leg and tap your heel on the floor. Then return to the starting position while avoiding touching the object. Perform 10 consecutive taps with the right leg. Then do the same task stepping with the left. Try to minimize the amount of body sway while tapping the step. Try to avoid allowing the water to spill. I will be here to guard you for safety.

**Verbal Cues for Feedback:**

- Remember to shift your weight so that your center of mass is over the stance foot.
- Try to minimize the amount that you sway.

**Successful Repetition:**

- Completes 10 step overs on each foot with no more than ankle strategy to maintain balance.
- Does not require use of support surface or physical assistance to maintain balance.
- No water spills.

**Unsuccessful Repetition:**

- Unable to complete 10 step overs on each foot AND/OR uses hip strategy or more to maintain balance.
- Requires use of support surface or physical assistance to maintain balance.
- Water spills.

**Performance:**

- **Set 1**
  - **Successful Repetitions: R:____/10____ L:____/10____**
  - **Number of Verbal Cues: R:__________ L:__________**
- **Set 2**
  - **Successful Repetitions: R:____/10____ L:____/10____**
  - **Number of Verbal Cues: R:__________ L:__________**
- **Set 3**
  - **Successful Repetitions: R:____/10____ L:____/10____**
  - **Number of Verbal Cues: R:__________ L:__________**

**Criteria for Progression:**

- **Successful performance of all repetitions and sets.**
- **No more than 2 verbal cues provided per set per side.**

**Does the participant meet the criteria for progression? YES / NO**

- **If YES, progress to LEVEL 8.**
- **If NO, repeat this exercise at next session.**

Anticipatory Postural Adjustments – Exercises

**Standing Step Up and Over Obstacle**

**Level 8: Standing Step Up and Over Small Obstacle WITHOUT UE Assistance while on FOAM WITH DUAL TASK**

**Equipment: Treadmill rail, chair, or wall for support AND foam roll (6 in. diameter).**

**Instructions:** I will ask you step up and over this obstacle, tap your heel on the floor, and return to the starting position. You will complete this task while standing on foam and holding a glass of water. Shift your weight to the stance leg and step up and over the obstacle with the opposite leg and tap your heel on the floor. Then return to the starting position while avoiding touching the object. Perform 10 consecutive taps with the right leg. Then do the same task stepping with the left. Try to minimize the amount of body sway while tapping the step. Try to avoid allowing the water to spill. I will be here to guard you for safety.

**Verbal Cues for Feedback:**

- Remember to shift your weight so that your center of mass is over the stance foot.
- Try to minimize the amount that you sway.

**Successful Repetition:**

- Completes 10 step overs on each foot with no more than ankle strategy to maintain balance.
- Does not require use of support surface or physical assistance to maintain balance.
- No water spills.

**Unsuccessful Repetition:**

- Unable to complete 10 step overs on each foot AND/OR uses hip strategy or more to maintain balance.
- Requires use of support surface or physical assistance to maintain balance.
- Water spills.

**Performance:**

- **Set 1**
  - **Successful Repetitions: R:____/10____ L:____/10____**
  - **Number of Verbal Cues: R:__________ L:__________**
- **Set 2**
  - **Successful Repetitions: R:____/10____ L:____/10____**
  - **Number of Verbal Cues: R:__________ L:__________**
- **Set 3**
  - **Successful Repetitions: R:____/10____ L:____/10____**
  - **Number of Verbal Cues: R:__________ L:__________**

**Criteria for Progression:**

- **Successful performance of all repetitions and sets.**
- **No more than 2 verbal cues provided per set per side.**

**Does the participant meet the criteria for progression? YES / NO**

- **If YES, continue this exercise for the remainder of your PT sessions.**
- **If NO, repeat this exercise at next session.**

**Postural Reponses**

**Perturbation Training (with producing a step) – Anterior/Posterior and Lateral Directions**

**Level 1: Standing on Firm Surface with Perturbation to Produce Stepping Response**

**Equipment:** Gait belt

**Instructions:** Please stand comfortably with your feet shoulder-width apart. I will give you a small push forward, backward, or sideways. This push will be enough to cause you to step. I would like to you to take as few steps as possible to catch your balance after being pushed. We will do 25 repetitions in each direction (forward, backward, left, right).

**Verbal Cues for Feedback:**

- Take as few steps as possible to catch your balance.

**Successful Repetition:**

- Catches balance without physical assistance.

**Unsuccessful Repetition:**

- Requires physical assistance to catch balance.

**Performance:**

- **Anterior**
  - **Successful Repetitions: _____/25____**
- **Posterior**
  - **Successful Repetitions: _____/25____**
- **Left**
  - **Successful Repetitions: _____/25____**
- **Right**
  - **Successful Repetitions: _____/25____**

**This exercise will be done at each session for the duration of the PT intervention.**

**Sensory Orientation**

**Semi-Tandem Stance, Eyes Open, Firm Surface**

**Level 1:** Standing – Semi-Tandem, Eyes Open, Firm Surface

**Equipment:** Chair, Railing

Instructions: I am going to ask you to stand in stride. You will keep your eyes open and try maintain your balance. Try to minimize the amount that you sway.

**Verbal Cues for Feedback:**

- Keep your feet in stride throughout the task.
- Try to stand up straight.
- Try to avoid bending your hips.

**Successful Repetition:**

- Completes 5 repetitions (30 seconds each) successfully without loss of balance (i.e. ≥ hip strategy).

**Unsuccessful Repetition:**

- Unable to complete repetition without loss of balance (i.e. ≥ hip strategy).

**Performance:**

- **Set 1**
  - **Successful Repetitions: ____/5____**
  - **Number of Verbal Cues: __________**

**Criteria for Progression**

- Successful performance of all repetitions.
- No more than 2 verbal cues provided per set.

**Does the participant meet the criteria for progression? YES / NO**

- If YES, progress to LEVEL 2 at next session.
- If NO, repeat this exercise at next session.

**Sensory Orientation**

**Semi-Tandem, Eyes Closed, Firm Surface**

**Level 2:** Standing – Semi-Tandem, Eyes Closed, Firm Surface

**Equipment:** Chair, Railing

Instructions: I am going to ask you to stand in stride. You will keep your eyes closed and try maintain your balance. I will tell you when to open your eyes. Try to minimize the amount that you sway.

**Verbal Cues for Feedback:**

- Keep your feet in place if possible.
- Try to stand up straight.
- Try to avoid bending your hips.
- Keep your eyes closed.

**Successful Repetition:**

- Completes 5 repetitions (30 seconds each) successfully without loss of balance (i.e. ≥ hip strategy).
- Maintains eyes closed.

**Unsuccessful Repetition:**

- Unable to complete repetition without loss of balance (i.e. ≥ hip strategy).
- Opens eyes.

**Performance:**

- **Set 1**
  - **Successful Repetitions: ____/5____**
  - **Number of Verbal Cues: __________**

**Criteria for Progression**

- Successful performance of all repetitions.
- No more than 2 verbal cues provided per set.

**Does the participant meet the criteria for progression? YES / NO**

- If YES, progress to LEVEL 3 at next session.
- If NO, repeat this exercise at next session.

**Sensory Orientation**

**Tandem Stance, Eyes Open, Firm Surface**

**Level 3:** Standing – Tandem Stance, Eyes Open, Firm Surface

**Equipment:** Chair, Railing

Instructions: I am going to ask you to stand in place with one foot directly in front of the other. You will feel like you’re standing on a tight rope. You will keep your eyes open and try maintain your balance. Try to minimize the amount that you sway.

**Verbal Cues for Feedback:**

- Keep your feet in place if possible.
- Try to stand up straight.
- Try to avoid allowing your hips to bend.

**Successful Repetition:**

- Completes 5 repetitions (30 seconds each) successfully without loss of balance (i.e. ≥ hip strategy).

**Unsuccessful Repetition:**

- Unable to complete repetition without loss of balance (i.e. ≥ hip strategy).

**Performance:**

- **Set 1**
  - **Successful Repetitions: ____/5____**
  - **Number of Verbal Cues: __________**

**Criteria for Progression**

- Successful performance of all repetitions.
- No more than 2 verbal cues provided per set.

**Does the participant meet the criteria for progression? YES / NO**

- If YES, progress to LEVEL 4 at next session.
- If NO, repeat this exercise at next session.

**Sensory Orientation**

**Tandem Stance, Eyes Closed, Firm Surface**

**Level 4:** Tandem Stance, Eyes Open, Foam Surface

**Equipment:** Chair, Railing

Instructions: I am going to ask you to stand in place with one foot directly in front of the other. You will feel like you’re standing on a tight rope. You will keep your eyes closed and try maintain your balance. I will tell you when to open your eyes. Try to minimize the amount that you sway.

**Verbal Cues for Feedback:**

- Keep your feet in place if possible.
- Try to stand up straight.
- Try to avoid bending your hips.
- Keep your eyes closed.

**Successful Repetition:**

- Completes 5 repetitions (30 seconds each) successfully without loss of balance (i.e. ≥ hip strategy).
- Maintains eyes closed.

**Unsuccessful Repetition:**

- Unable to complete repetition without loss of balance (i.e. ≥ hip strategy).
- Opens eyes.

**Performance:**

- **Set 1**
  - **Successful Repetitions: ____/5____**
  - **Number of Verbal Cues: __________**

**Criteria for Progression**

- Successful performance of all repetitions.
- No more than 2 verbal cues provided per set.

**Does the participant meet the criteria for progression? YES / NO**

- If YES, progress to LEVEL 5 at next session.
- If NO, repeat this exercise at next session.

**Sensory Orientation**

**Tandem Stance, Eyes Open, Foam Surface**

**Level 5:** Standing – Tandem Stance, Eyes Open, Foam Surface

**Equipment:** Chair, Railing, Foam Surface

Instructions: I am going to ask you to stand in place with one foot directly in front of the other while on this foam block. You will feel like you’re standing on a tight rope. You will keep your eyes open and try maintain your balance. Try to minimize the amount that you sway.

**Verbal Cues for Feedback:**

- Keep your feet in place if possible.
- Try to stand up straight.
- Try to avoid allowing your hips to bend.

**Successful Repetition:**

- Completes 5 repetitions (30 seconds each) successfully without loss of balance (i.e. ≥ hip strategy).

**Unsuccessful Repetition:**

- Unable to complete repetition without loss of balance (i.e. ≥ hip strategy).

**Performance:**

- **Set 1**
  - **Successful Repetitions: ____/5____**
  - **Number of Verbal Cues: __________**

**Criteria for Progression**

- Successful performance of all repetitions.
- No more than 2 verbal cues provided per set.

**Does the participant meet the criteria for progression? YES / NO**

- If YES, progress to LEVEL 6 at next session.
- If NO, repeat this exercise at next session.

**Sensory Orientation**

**Tandem Stance, Eyes Closed, Foam Surface**

**Level 6:** Standing – Tandem Stance, Eyes Open, Foam Surface

**Equipment:** Chair, Railing, Foam Surface

Instructions: I am going to ask you to stand in place with one foot directly in front of the other while on this foam block. You will feel like you’re standing on a tight rope. You will keep your eyes closed and try maintain your balance. I will tell you when to open your eyes. Try to minimize the amount that you sway.

**Verbal Cues for Feedback:**

- Keep your feet in place if possible.
- Try to stand up straight.
- Try to avoid bending your hips.
- Keep your eyes closed.

**Successful Repetition:**

- Completes 5 repetitions (30 seconds each) successfully without loss of balance (i.e. ≥ hip strategy).
- Maintains eyes closed.

**Unsuccessful Repetition:**

- Unable to complete repetition without loss of balance (i.e. ≥ hip strategy).
- Opens eyes.

**Performance:**

- **Set 1**
  - **Successful Repetitions: ____/5____**
  - **Number of Verbal Cues: __________**

**Criteria for Progression**

- Successful performance of all repetitions.
- No more than 2 verbal cues provided per set.

**Does the participant meet the criteria for progression? YES / NO**

- If YES, progress to LEVEL 7 at next session.
- If NO, repeat this exercise at next session.

**Sensory Orientation**

**Tandem Stance, Eyes Open, Theraband Stability Trainer Pads (Advanced Level Black Inflatable Pads)**

**Level 7:** Standing – Tandem Stance, Eyes Open, Theraband Stability Trainer Pads (Advanced Level Black Inflatable Pads)

**Equipment:** Chair, Railing, Theraband Stability Trainer Pads (Advanced Level Black Inflatable Pads)

Instructions: I am going to ask you to stand in place with one foot directly in front of the other with one foot on each of these Theraband Stability Trainer Pads (Advanced Level Black Inflatable Pads). You will feel like you’re standing on a tight rope. You will keep your eyes open and try maintain your balance. Try to minimize the amount that you sway.

**Verbal Cues for Feedback:**

- Keep your feet in place if possible.
- Try to stand up straight.
- Try to avoid allowing your hips to bend.

**Successful Repetition:**

- Completes 5 repetitions (30 seconds each) successfully without loss of balance (i.e. ≥ hip strategy).

**Unsuccessful Repetition:**

- Unable to complete repetition without loss of balance (i.e. ≥ hip strategy).

**Performance:**

- **Set 1**
  - **Successful Repetitions: ____/5____**
  - **Number of Verbal Cues: __________**

**Criteria for Progression**

- Successful performance of all repetitions.
- No more than 2 verbal cues provided per set.

**Does the participant meet the criteria for progression? YES / NO**

- If YES, progress to LEVEL 8 at next session.
- If NO, repeat this exercise at next session.

**Sensory Orientation**

**Tandem Stance, Eyes Closed, Theraband Stability Trainer Pads (Advanced Level Black Inflatable Pads)**

**Level 8:** Standing – Tandem Stance, Eyes Closed, Theraband Stability Trainer Pads (Advanced Level Black Inflatable Pads)

**Equipment:** Chair, Railing, Theraband Stability Trainer Pads (Advanced Level Black Inflatable Pads)

Instructions: I am going to ask you to stand in place with one foot directly in front of the other with one foot on each of these Theraband Stability Trainer Pads (Advanced Level Black Inflatable Pads). You will feel like you’re standing on a tight rope. You will keep your eyes closed and try maintain your balance. I will tell you when to open your eyes. Try to minimize the amount that you sway.

**Verbal Cues for Feedback:**

- Keep your feet in place if possible.
- Try to stand up straight.
- Try to avoid bending your hips.
- Keep your eyes closed.

**Successful Repetition:**

- Completes 5 repetitions (30 seconds each) successfully without loss of balance (i.e. ≥ hip strategy).
- Maintains eyes closed.

**Unsuccessful Repetition:**

- Unable to complete repetition without loss of balance (i.e. ≥ hip strategy).
- Opens eyes.

**Performance:**

- **Set 1**
  - **Successful Repetitions: ____/5____**
  - **Number of Verbal Cues: __________**

**Criteria for Progression**

- Successful performance of all repetitions.
- No more than 2 verbal cues provided per set.

**Does the participant meet the criteria for progression? YES / NO**

- If YES, progress to LEVEL 9 at next session.
- If NO, repeat this exercise at next session.

**Sensory Orientation**

**Tandem Stance, Eyes Open, Theraband Stability Trainer Pads (Advanced Level Black Inflatable Pads), Dual Task**

**Level 9:** Standing – Tandem Stance, Eyes Open, Theraband Stability Trainer Pads (Advanced Level Black Inflatable Pads), Dual Task

**Equipment:** Chair, Railing, Theraband Stability Trainer Pads (Advanced Level Black Inflatable Pads), Cup of Water

Instructions: I am going to ask you to stand in place with one foot directly in front of the other with one foot on each of these Theraband Stability Trainer Pads (Advanced Level Black Inflatable Pads). You will feel like you’re standing on a tight rope. While doing this, you will hold this cup of water and try not to let it spill. You will keep your eyes open and try maintain your balance. Try to minimize the amount that you sway.

**Verbal Cues for Feedback:**

- Keep your feet in place if possible.
- Try to stand up straight.
- Try to avoid allowing your hips to bend.
- Try not to let the water spill.

**Successful Repetition:**

- Completes 5 repetitions (30 seconds each) successfully without loss of balance (i.e. ≥ hip strategy).

**Unsuccessful Repetition:**

- Unable to complete repetition without loss of balance (i.e. ≥ hip strategy).
- Water spills.

**Performance:**

- **Set 1**
  - **Successful Repetitions: ____/5____**
  - **Number of Verbal Cues: __________**

**Criteria for Progression**

- Successful performance of all repetitions.
- No more than 2 verbal cues provided per set.

**Does the participant meet the criteria for progression? YES / NO**

- If YES, progress to LEVEL 10 at next session.
- If NO, repeat this exercise at next session.

**Sensory Orientation**

**Tandem Stance, Eyes Closed, Theraband Stability Trainer Pads (Advanced Level Black Inflatable Pads), Dual Task**

**Level 10:** Standing – Tandem Stance, Eyes Closed, Theraband Stability Trainer Pads (Advanced Level Black Inflatable Pads), Dual Task

**Equipment:** Chair, Railing, Theraband Stability Trainer Pads (Advanced Level Black Inflatable Pads), Cup of Water

Instructions: I am going to ask you to stand in place with one foot directly in front of the other with one foot on each of these Theraband Stability Trainer Pads (Advanced Level Black Inflatable Pads). You will feel like you’re standing on a tight rope. While doing this, you will close your eyes and hold this cup of water and try not to let it spill while maintaining your balance. I will tell you when to open your eyes. Try to minimize the amount that you sway.

**Verbal Cues for Feedback:**

- Keep your feet in place if possible.
- Try to stand up straight.
- Try to avoid allowing your hips to bend.
- Try not to let the water spill.

**Successful Repetition:**

- Completes 5 repetitions (30 seconds each) successfully without loss of balance (i.e. ≥ hip strategy).

**Unsuccessful Repetition:**

- Unable to complete repetition without loss of balance (i.e. ≥ hip strategy).
- Water spills.

**Performance:**

- **Set 1**
  - **Successful Repetitions: ____/5____**
  - **Number of Verbal Cues: __________**

**Criteria for Progression**

- Successful performance of all repetitions.
- No more than 2 verbal cues provided per set.

**Does the participant meet the criteria for progression? YES / NO**

- If YES, continue this exercise for the remainder of your PT sessions.
- If NO, repeat this exercise at next session.

**Stability in Gait**

**Treadmill Walking**

**Week 1, Visit 1:** Walking at pace 10% above comfortable overground pace.

**Equipment:** Treadmill

**PT Instructions:** Administer the 10 Meter Walk Test to calculate overground gait velocity. The participant should be instructed to walk at their comfortable pace. The PT should first orient the participant to the treadmill showing them how to stop it if necessary. The participant should wear the emergency shut off band. Demonstrate what is meant by a good heel strike. If you notice 3 consecutive steps in which the feet scuff the treadmill, provide verbal cue. Place a gait belt on the participant. Stand behind the participant on the treadmill platform with your hands near the participant’s waist to guard for safety.

**Instructions:** I will ask you to talk on the treadmill for 10 minutes at a pace that is slightly above your comfortable pace. If at any point you feel you are short of breath or are unable to continue, please tell the physical therapist or push the red stop button. Please focus on taking consistent strides with a good heel strike at each step. You may hold onto the railings if this helps you feel more stable.

**Comfortable Overground Pace: __________________m/sec.**

**Comfortable Overground Pace: __________________mph.**

**Treadmill Walking Pace:**

**Calculation: (Comfortable Overground Pace in MPH x 0.10) + Comfortable Overgound Pace in MPH. Round to nearest 10^th^ of a decimal point.**

**10% Above Comfortable Overground Pace: ___________________mph (Round to the nearest 10^th^)**

**Verbal Cues for Feedback:**

- Continue focusing on good heel strike with each step.
- Try to stand up straight as you walk.

**Successful Repetition:**

- Completes 10 minutes of treadmill walking with no more than 10 verbal cues.

**Unsuccessful Repetition:**

- Unable to complete 10 minutes of treadmill walking.
- Requires more than 10 verbal cues over the 10 minutes.

**Did you have to adjust the pace of the treadmill? YES / NO**

**If YES, at which time point did you adjust the pace? ________________**

**If YES, to which speed did you set the treadmill? ________________mph.**

**If YES, was the participant able to finish the treadmill session at this pace? YES / NO**

**Stability in Gait**

**Treadmill Walking**

**Week 1, Visit 2:** Walking at pace 10% above comfortable overground pace.

**Equipment:** Treadmill

**PT Instructions:** The PT should first orient the participant to the treadmill showing them how to stop it if necessary. The participant should wear the emergency shut off band. Demonstrate what is meant by a good heel strike. If you notice 3 consecutive steps in which the feet scuff the treadmill, provide verbal cue. Place a gait belt on the participant. Stand behind the participant on the treadmill platform with your hands near the participant’s waist to guard for safety.

**Instructions:** I will ask you to talk on the treadmill for 10 minutes at a pace that is slightly above your comfortable pace. If at any point you feel you are short of breath or are unable to continue, please tell the physical therapist or push the red stop button. Please focus on taking consistent strides with a good heel strike at each step. You may hold onto the railings if this helps you feel more stable.

**Treadmill Walking Pace:**

**10% Above Comfortable Overground Pace (take from Week 1, Visit 1): ___________________mph**

**Verbal Cues for Feedback:**

- Continue focusing on good heel strike with each step.
- Try to stand up straight as you walk.

**Successful Repetition:**

- Completes 10 minutes of treadmill walking with no more than 10 verbal cues.

**Unsuccessful Repetition:**

- Unable to complete 10 minutes of treadmill walking.
- Requires more than 10 verbal cues over the 10 minutes.

**Did you have to adjust the pace of the treadmill? YES / NO**

**If YES, at which time point did you adjust the pace? ________________**

**If YES, to which speed did you set the treadmill? ________________mph.**

**If YES, was the participant able to finish the treadmill session at this pace? YES / NO**

**Stability in Gait**

**Treadmill Walking**

**Week 2, Visit 1:** Walking at pace 10% above comfortable overground pace.

**Equipment:** Treadmill

**PT Instructions:** Administer the 10 Meter Walk Test to calculate overground gait velocity. The participant should be instructed to walk at their comfortable pace. The PT should first orient the participant to the treadmill showing them how to stop it if necessary. The participant should wear the emergency shut off band. Demonstrate what is meant by a good heel strike. If you notice 3 consecutive steps in which the feet scuff the treadmill, provide verbal cue. Place a gait belt on the participant. Stand behind the participant on the treadmill platform with your hands near the participant’s waist to guard for safety.

**Instructions:** I will ask you to talk on the treadmill for 10 minutes at a pace that is slightly above your comfortable pace. If at any point you feel you are short of breath or are unable to continue, please tell the physical therapist or push the red stop button. Please focus on taking consistent strides with a good heel strike at each step. You may hold onto the railings if this helps you feel more stable.

**Comfortable Overground Pace: __________________m/sec.**

**Comfortable Overground Pace: __________________mph.**

**Treadmill Walking Pace:**

**Calculation: (Comfortable Overground Pace in MPH x 0.10) + Comfortable Overgound Pace in MPH. Round to nearest 10^th^ of a decimal point.**

**10% Above Comfortable Overground Pace: ___________________mph (Round to the nearest 10^th^)**

******If this pace is lower than previous week’s pace, use the highest recorded pace******

**Week 2, Visit 1 Treadmill Pace: _____________________mph.**

**Verbal Cues for Feedback:**

- Continue focusing on good heel strike with each step.
- Try to stand up straight as you walk.

**Successful Repetition:**

- Completes 10 minutes of treadmill walking with no more than 10 verbal cues.

**Unsuccessful Repetition:**

- Unable to complete 10 minutes of treadmill walking.
- Requires more than 10 verbal cues over the 10 minutes.

**Did you have to adjust the pace of the treadmill? YES / NO**

**If YES, at which time point did you adjust the pace? ________________**

**If YES, to which speed did you set the treadmill? ________________mph.**

**If YES, was the participant able to finish the treadmill session at this pace? YES / NO**

**Stability in Gait**

**Treadmill Walking**

**Week 2, Visit 2:** Walking at pace 10% above comfortable overground pace.

**Equipment:** Treadmill

**PT Instructions:** The PT should first orient the participant to the treadmill showing them how to stop it if necessary. The participant should wear the emergency shut off band. Demonstrate what is meant by a good heel strike. If you notice 3 consecutive steps in which the feet scuff the treadmill, provide verbal cue. Place a gait belt on the participant. Stand behind the participant on the treadmill platform with your hands near the participant’s waist to guard for safety.

**Instructions:** I will ask you to talk on the treadmill for 10 minutes at a pace that is slightly above your comfortable pace. If at any point you feel you are short of breath or are unable to continue, please tell the physical therapist or push the red stop button. Please focus on taking consistent strides with a good heel strike at each step. You may hold onto the railings if this helps you feel more stable.

**Treadmill Walking Pace:**

**10% Above Comfortable Overground Pace (take from Week 2, Visit 1): ___________________mph**

**Verbal Cues for Feedback:**

- Continue focusing on good heel strike with each step.
- Try to stand up straight as you walk.

**Successful Repetition:**

- Completes 10 minutes of treadmill walking with no more than 10 verbal cues.

**Unsuccessful Repetition:**

- Unable to complete 10 minutes of treadmill walking.
- Requires more than 10 verbal cues over the 10 minutes.

**Did you have to adjust the pace of the treadmill? YES / NO**

**If YES, at which time point did you adjust the pace? ________________**

**If YES, to which speed did you set the treadmill? ________________mph.**

**If YES, was the participant able to finish the treadmill session at this pace? YES / NO**

**Stability in Gait**

**Treadmill Walking**

**Week 3, Visit 1:** Walking at pace 10% above comfortable overground pace.

**Equipment:** Treadmill

**PT Instructions:** Administer the 10 Meter Walk Test to calculate overground gait velocity. The participant should be instructed to walk at their comfortable pace. The PT should first orient the participant to the treadmill showing them how to stop it if necessary. The participant should wear the emergency shut off band. Demonstrate what is meant by a good heel strike. If you notice 3 consecutive steps in which the feet scuff the treadmill, provide verbal cue. Place a gait belt on the participant. Stand behind the participant on the treadmill platform with your hands near the participant’s waist to guard for safety.

**Instructions:** I will ask you to talk on the treadmill for 10 minutes at a pace that is slightly above your comfortable pace. If at any point you feel you are short of breath or are unable to continue, please tell the physical therapist or push the red stop button. Please focus on taking consistent strides with a good heel strike at each step. You may hold onto the railings if this helps you feel more stable.

**Comfortable Overground Pace: __________________m/sec.**

**Comfortable Overground Pace: __________________mph.**

**Treadmill Walking Pace:**

**Calculation: (Comfortable Overground Pace in MPH x 0.10) + Comfortable Overgound Pace in MPH. Round to nearest 10^th^ of a decimal point.**

**10% Above Comfortable Overground Pace: ___________________mph (Round to the nearest 10^th^)**

******If this pace is lower than previous week’s pace, use the highest recorded pace******

**Week 3, Visit 1 Treadmill Pace: _____________________mph.**

**Verbal Cues for Feedback:**

- Continue focusing on good heel strike with each step.
- Try to stand up straight as you walk.

**Successful Repetition:**

- Completes 10 minutes of treadmill walking with no more than 10 verbal cues.

**Unsuccessful Repetition:**

- Unable to complete 10 minutes of treadmill walking.
- Requires more than 10 verbal cues over the 10 minutes.

**Did you have to adjust the pace of the treadmill? YES / NO**

**If YES, at which time point did you adjust the pace? ________________**

**If YES, to which speed did you set the treadmill? ________________mph.**

**If YES, was the participant able to finish the treadmill session at this pace? YES / NO**

**Stability in Gait**

**Treadmill Walking**

**Week 3, Visit 2:** Walking at pace 10% above comfortable overground pace.

**Equipment:** Treadmill

**PT Instructions:** The PT should first orient the participant to the treadmill showing them how to stop it if necessary. The participant should wear the emergency shut off band. Demonstrate what is meant by a good heel strike. If you notice 3 consecutive steps in which the feet scuff the treadmill, provide verbal cue. Place a gait belt on the participant. Stand behind the participant on the treadmill platform with your hands near the participant’s waist to guard for safety.

**Instructions:** I will ask you to talk on the treadmill for 10 minutes at a pace that is slightly above your comfortable pace. If at any point you feel you are short of breath or are unable to continue, please tell the physical therapist or push the red stop button. Please focus on taking consistent strides with a good heel strike at each step. You may hold onto the railings if this helps you feel more stable.

**Treadmill Walking Pace:**

**10% Above Comfortable Overground Pace (take from Week 3, Visit 1): ___________________mph**

**Verbal Cues for Feedback:**

- Continue focusing on good heel strike with each step.
- Try to stand up straight as you walk.

**Successful Repetition:**

- Completes 10 minutes of treadmill walking with no more than 10 verbal cues.

**Unsuccessful Repetition:**

- Unable to complete 10 minutes of treadmill walking.
- Requires more than 10 verbal cues over the 10 minutes.

**Did you have to adjust the pace of the treadmill? YES / NO**

**If YES, at which time point did you adjust the pace? ________________**

**If YES, to which speed did you set the treadmill? ________________mph.**

**If YES, was the participant able to finish the treadmill session at this pace? YES / NO**

**Stability in Gait**

**Treadmill Walking**

**Week 4, Visit 1:** Walking at pace 10% above comfortable overground pace.

**Equipment:** Treadmill

**PT Instructions:** Administer the 10 Meter Walk Test to calculate overground gait velocity. The participant should be instructed to walk at their comfortable pace. The PT should first orient the participant to the treadmill showing them how to stop it if necessary. The participant should wear the emergency shut off band. Demonstrate what is meant by a good heel strike. If you notice 3 consecutive steps in which the feet scuff the treadmill, provide verbal cue. Place a gait belt on the participant. Stand behind the participant on the treadmill platform with your hands near the participant’s waist to guard for safety.

**Instructions:** I will ask you to talk on the treadmill for 10 minutes at a pace that is slightly above your comfortable pace. If at any point you feel you are short of breath or are unable to continue, please tell the physical therapist or push the red stop button. Please focus on taking consistent strides with a good heel strike at each step. You may hold onto the railings if this helps you feel more stable.

**Comfortable Overground Pace: __________________m/sec.**

**Comfortable Overground Pace: __________________mph.**

**Treadmill Walking Pace:**

**Calculation: (Comfortable Overground Pace in MPH x 0.10) + Comfortable Overgound Pace in MPH. Round to nearest 10^th^ of a decimal point.**

**10% Above Comfortable Overground Pace: ___________________mph (Round to the nearest 10^th^)**

******If this pace is lower than previous week’s pace, use the highest recorded pace******

**Week 4, Visit 1 Treadmill Pace: _____________________mph.**

**Verbal Cues for Feedback:**

- Continue focusing on good heel strike with each step.
- Try to stand up straight as you walk.

**Successful Repetition:**

- Completes 10 minutes of treadmill walking with no more than 10 verbal cues.

**Unsuccessful Repetition:**

- Unable to complete 10 minutes of treadmill walking.
- Requires more than 10 verbal cues over the 10 minutes.

**Did you have to adjust the pace of the treadmill? YES / NO**

**If YES, at which time point did you adjust the pace? ________________**

**If YES, to which speed did you set the treadmill? ________________mph.**

**If YES, was the participant able to finish the treadmill session at this pace? YES / NO**

**Stability in Gait**

**Treadmill Walking**

**Week 4, Visit 2:** Walking at pace 10% above comfortable overground pace.

**Equipment:** Treadmill

**PT Instructions:** The PT should first orient the participant to the treadmill showing them how to stop it if necessary. The participant should wear the emergency shut off band. Demonstrate what is meant by a good heel strike. If you notice 3 consecutive steps in which the feet scuff the treadmill, provide verbal cue. Place a gait belt on the participant. Stand behind the participant on the treadmill platform with your hands near the participant’s waist to guard for safety.

**Instructions:** I will ask you to talk on the treadmill for 10 minutes at a pace that is slightly above your comfortable pace. If at any point you feel you are short of breath or are unable to continue, please tell the physical therapist or push the red stop button. Please focus on taking consistent strides with a good heel strike at each step. You may hold onto the railings if this helps you feel more stable.

**Treadmill Walking Pace:**

**10% Above Comfortable Overground Pace (take from Week 4, Visit 1): ___________________mph**

**Verbal Cues for Feedback:**

- Continue focusing on good heel strike with each step.
- Try to stand up straight as you walk.

**Successful Repetition:**

- Completes 10 minutes of treadmill walking with no more than 10 verbal cues.

**Unsuccessful Repetition:**

- Unable to complete 10 minutes of treadmill walking.
- Requires more than 10 verbal cues over the 10 minutes.

**Did you have to adjust the pace of the treadmill? YES / NO**

**If YES, at which time point did you adjust the pace? ________________**

**If YES, to which speed did you set the treadmill? ________________mph.**

**If YES, was the participant able to finish the treadmill session at this pace? YES / NO**

**Stability in Gait**

**Treadmill Walking**

**Week 5, Visit 1:** Walking at pace 10% above comfortable overground pace.

**Equipment:** Treadmill

**PT Instructions:** Administer the 10 Meter Walk Test to calculate overground gait velocity. The participant should be instructed to walk at their comfortable pace. The PT should first orient the participant to the treadmill showing them how to stop it if necessary. The participant should wear the emergency shut off band. Demonstrate what is meant by a good heel strike. If you notice 3 consecutive steps in which the feet scuff the treadmill, provide verbal cue. Place a gait belt on the participant. Stand behind the participant on the treadmill platform with your hands near the participant’s waist to guard for safety.

**Instructions:** I will ask you to talk on the treadmill for 10 minutes at a pace that is slightly above your comfortable pace. If at any point you feel you are short of breath or are unable to continue, please tell the physical therapist or push the red stop button. Please focus on taking consistent strides with a good heel strike at each step. You may hold onto the railings if this helps you feel more stable.

**Comfortable Overground Pace: __________________m/sec.**

**Comfortable Overground Pace: __________________mph.**

**Treadmill Walking Pace:**

**Calculation: (Comfortable Overground Pace in MPH x 0.10) + Comfortable Overgound Pace in MPH. Round to nearest 10^th^ of a decimal point.**

**10% Above Comfortable Overground Pace: ___________________mph (Round to the nearest 10^th^)**

******If this pace is lower than previous week’s pace, use the highest recorded pace******

**Week 5, Visit 1 Treadmill Pace: _____________________mph.**

**Verbal Cues for Feedback:**

- Continue focusing on good heel strike with each step.
- Try to stand up straight as you walk.

**Successful Repetition:**

- Completes 10 minutes of treadmill walking with no more than 10 verbal cues.

**Unsuccessful Repetition:**

- Unable to complete 10 minutes of treadmill walking.
- Requires more than 10 verbal cues over the 10 minutes.

**Did you have to adjust the pace of the treadmill? YES / NO**

**If YES, at which time point did you adjust the pace? ________________**

**If YES, to which speed did you set the treadmill? ________________mph.**

**If YES, was the participant able to finish the treadmill session at this pace? YES / NO**

**Stability in Gait**

**Treadmill Walking**

**Week 5, Visit 2:** Walking at pace 10% above comfortable overground pace.

**Equipment:** Treadmill

**PT Instructions:** The PT should first orient the participant to the treadmill showing them how to stop it if necessary. The participant should wear the emergency shut off band. Demonstrate what is meant by a good heel strike. If you notice 3 consecutive steps in which the feet scuff the treadmill, provide verbal cue. Place a gait belt on the participant. Stand behind the participant on the treadmill platform with your hands near the participant’s waist to guard for safety.

**Instructions:** I will ask you to talk on the treadmill for 10 minutes at a pace that is slightly above your comfortable pace. If at any point you feel you are short of breath or are unable to continue, please tell the physical therapist or push the red stop button. Please focus on taking consistent strides with a good heel strike at each step. You may hold onto the railings if this helps you feel more stable.

**Treadmill Walking Pace:**

**10% Above Comfortable Overground Pace (take from Week 5, Visit 1): ___________________mph**

**Verbal Cues for Feedback:**

- Continue focusing on good heel strike with each step.
- Try to stand up straight as you walk.

**Successful Repetition:**

- Completes 10 minutes of treadmill walking with no more than 10 verbal cues.

**Unsuccessful Repetition:**

- Unable to complete 10 minutes of treadmill walking.
- Requires more than 10 verbal cues over the 10 minutes.

**Did you have to adjust the pace of the treadmill? YES / NO**

**If YES, at which time point did you adjust the pace? ________________**

**If YES, to which speed did you set the treadmill? ________________mph.**

**If YES, was the participant able to finish the treadmill session at this pace? YES / NO**

**Stability in Gait**

**Treadmill Walking**

**Week 6, Visit 1:** Walking at pace 10% above comfortable overground pace.

**Equipment:** Treadmill

**PT Instructions:** Administer the 10 Meter Walk Test to calculate overground gait velocity. The participant should be instructed to walk at their comfortable pace. The PT should first orient the participant to the treadmill showing them how to stop it if necessary. The participant should wear the emergency shut off band. Demonstrate what is meant by a good heel strike. If you notice 3 consecutive steps in which the feet scuff the treadmill, provide verbal cue. Place a gait belt on the participant. Stand behind the participant on the treadmill platform with your hands near the participant’s waist to guard for safety.

**Instructions:** I will ask you to talk on the treadmill for 10 minutes at a pace that is slightly above your comfortable pace. If at any point you feel you are short of breath or are unable to continue, please tell the physical therapist or push the red stop button. Please focus on taking consistent strides with a good heel strike at each step. You may hold onto the railings if this helps you feel more stable.

**Comfortable Overground Pace: __________________m/sec.**

**Comfortable Overground Pace: __________________mph.**

**Treadmill Walking Pace:**

**Calculation: (Comfortable Overground Pace in MPH x 0.10) + Comfortable Overgound Pace in MPH. Round to nearest 10^th^ of a decimal point.**

**10% Above Comfortable Overground Pace: ___________________mph (Round to the nearest 10^th^)**

******If this pace is lower than previous week’s pace, use the highest recorded pace******

**Week 6, Visit 1 Treadmill Pace: _____________________mph.**

**Verbal Cues for Feedback:**

- Continue focusing on good heel strike with each step.
- Try to stand up straight as you walk.

**Successful Repetition:**

- Completes 10 minutes of treadmill walking with no more than 10 verbal cues.

**Unsuccessful Repetition:**

- Unable to complete 10 minutes of treadmill walking.
- Requires more than 10 verbal cues over the 10 minutes.

**Did you have to adjust the pace of the treadmill? YES / NO**

**If YES, at which time point did you adjust the pace? ________________**

**If YES, to which speed did you set the treadmill? ________________mph.**

**If YES, was the participant able to finish the treadmill session at this pace? YES / NO**

**Stability in Gait**

**Treadmill Walking**

**Week 6, Visit 2:** Walking at pace 10% above comfortable overground pace.

**Equipment:** Treadmill

**PT Instructions:** The PT should first orient the participant to the treadmill showing them how to stop it if necessary. The participant should wear the emergency shut off band. Demonstrate what is meant by a good heel strike. If you notice 3 consecutive steps in which the feet scuff the treadmill, provide verbal cue. Place a gait belt on the participant. Stand behind the participant on the treadmill platform with your hands near the participant’s waist to guard for safety.

**Instructions:** I will ask you to talk on the treadmill for 10 minutes at a pace that is slightly above your comfortable pace. If at any point you feel you are short of breath or are unable to continue, please tell the physical therapist or push the red stop button. Please focus on taking consistent strides with a good heel strike at each step. You may hold onto the railings if this helps you feel more stable.

**Treadmill Walking Pace:**

**10% Above Comfortable Overground Pace (take from Week 6, Visit 1): ___________________mph**

**Verbal Cues for Feedback:**

- Continue focusing on good heel strike with each step.
- Try to stand up straight as you walk.

**Successful Repetition:**

- Completes 10 minutes of treadmill walking with no more than 10 verbal cues.

**Unsuccessful Repetition:**

- Unable to complete 10 minutes of treadmill walking.
- Requires more than 10 verbal cues over the 10 minutes.

**Did you have to adjust the pace of the treadmill? YES / NO**

**If YES, at which time point did you adjust the pace? ________________**

**If YES, to which speed did you set the treadmill? ________________mph.**

**If YES, was the participant able to finish the treadmill session at this pace? YES / NO**

**Stability in Gait**

**Treadmill Walking**

**Week 7, Visit 1:** Walking at pace 10% above comfortable overground pace.

**Equipment:** Treadmill

**PT Instructions:** Administer the 10 Meter Walk Test to calculate overground gait velocity. The participant should be instructed to walk at their comfortable pace. The PT should first orient the participant to the treadmill showing them how to stop it if necessary. The participant should wear the emergency shut off band. Demonstrate what is meant by a good heel strike. If you notice 3 consecutive steps in which the feet scuff the treadmill, provide verbal cue. Place a gait belt on the participant. Stand behind the participant on the treadmill platform with your hands near the participant’s waist to guard for safety.

**Instructions:** I will ask you to talk on the treadmill for 10 minutes at a pace that is slightly above your comfortable pace. If at any point you feel you are short of breath or are unable to continue, please tell the physical therapist or push the red stop button. Please focus on taking consistent strides with a good heel strike at each step. You may hold onto the railings if this helps you feel more stable.

**Comfortable Overground Pace: __________________m/sec.**

**Comfortable Overground Pace: __________________mph.**

**Treadmill Walking Pace:**

**Calculation: (Comfortable Overground Pace in MPH x 0.10) + Comfortable Overgound Pace in MPH. Round to nearest 10^th^ of a decimal point.**

**10% Above Comfortable Overground Pace: ___________________mph (Round to the nearest 10^th^)**

******If this pace is lower than previous week’s pace, use the highest recorded pace******

**Week 7, Visit 1 Treadmill Pace: _____________________mph.**

**Verbal Cues for Feedback:**

- Continue focusing on good heel strike with each step.
- Try to stand up straight as you walk.

**Successful Repetition:**

- Completes 10 minutes of treadmill walking with no more than 10 verbal cues.

**Unsuccessful Repetition:**

- Unable to complete 10 minutes of treadmill walking.
- Requires more than 10 verbal cues over the 10 minutes.

**Did you have to adjust the pace of the treadmill? YES / NO**

**If YES, at which time point did you adjust the pace? ________________**

**If YES, to which speed did you set the treadmill? ________________mph.**

**If YES, was the participant able to finish the treadmill session at this pace? YES / NO**

**Stability in Gait**

**Treadmill Walking**

**Week 7, Visit 2:** Walking at pace 10% above comfortable overground pace.

**Equipment:** Treadmill

**PT Instructions:** The PT should first orient the participant to the treadmill showing them how to stop it if necessary. The participant should wear the emergency shut off band. Demonstrate what is meant by a good heel strike. If you notice 3 consecutive steps in which the feet scuff the treadmill, provide verbal cue. Place a gait belt on the participant. Stand behind the participant on the treadmill platform with your hands near the participant’s waist to guard for safety.

**Instructions:** I will ask you to talk on the treadmill for 10 minutes at a pace that is slightly above your comfortable pace. If at any point you feel you are short of breath or are unable to continue, please tell the physical therapist or push the red stop button. Please focus on taking consistent strides with a good heel strike at each step. You may hold onto the railings if this helps you feel more stable.

**Treadmill Walking Pace:**

**10% Above Comfortable Overground Pace (take from Week 7, Visit 1): ___________________mph**

**Verbal Cues for Feedback:**

- Continue focusing on good heel strike with each step.
- Try to stand up straight as you walk.

**Successful Repetition:**

- Completes 10 minutes of treadmill walking with no more than 10 verbal cues.

**Unsuccessful Repetition:**

- Unable to complete 10 minutes of treadmill walking.
- Requires more than 10 verbal cues over the 10 minutes.

**Did you have to adjust the pace of the treadmill? YES / NO**

**If YES, at which time point did you adjust the pace? ________________**

**If YES, to which speed did you set the treadmill? ________________mph.**

**If YES, was the participant able to finish the treadmill session at this pace? YES / NO**

**Stability in Gait**

**Treadmill Walking**

**Week 8, Visit 1:** Walking at pace 10% above comfortable overground pace.

**Equipment:** Treadmill

**PT Instructions:** Administer the 10 Meter Walk Test to calculate overground gait velocity. The participant should be instructed to walk at their comfortable pace. The PT should first orient the participant to the treadmill showing them how to stop it if necessary. The participant should wear the emergency shut off band. Demonstrate what is meant by a good heel strike. If you notice 3 consecutive steps in which the feet scuff the treadmill, provide verbal cue. Place a gait belt on the participant. Stand behind the participant on the treadmill platform with your hands near the participant’s waist to guard for safety.

**Instructions:** I will ask you to talk on the treadmill for 10 minutes at a pace that is slightly above your comfortable pace. If at any point you feel you are short of breath or are unable to continue, please tell the physical therapist or push the red stop button. Please focus on taking consistent strides with a good heel strike at each step. You may hold onto the railings if this helps you feel more stable.

**Comfortable Overground Pace: __________________m/sec.**

**Comfortable Overground Pace: __________________mph.**

**Treadmill Walking Pace:**

**Calculation: (Comfortable Overground Pace in MPH x 0.10) + Comfortable Overgound Pace in MPH. Round to nearest 10^th^ of a decimal point.**

**10% Above Comfortable Overground Pace: ___________________mph (Round to the nearest 10^th^)**

******If this pace is lower than previous week’s pace, use the highest recorded pace******

**Week 8, Visit 1 Treadmill Pace: _____________________mph.**

**Verbal Cues for Feedback:**

- Continue focusing on good heel strike with each step.
- Try to stand up straight as you walk.

**Successful Repetition:**

- Completes 10 minutes of treadmill walking with no more than 10 verbal cues.

**Unsuccessful Repetition:**

- Unable to complete 10 minutes of treadmill walking.
- Requires more than 10 verbal cues over the 10 minutes.

**Did you have to adjust the pace of the treadmill? YES / NO**

**If YES, at which time point did you adjust the pace? ________________**

**If YES, to which speed did you set the treadmill? ________________mph.**

**If YES, was the participant able to finish the treadmill session at this pace? YES / NO**

**Stability in Gait**

**Treadmill Walking**

**Week 8, Visit 2:** Walking at pace 10% above comfortable overground pace.

**Equipment:** Treadmill

**PT Instructions:** The PT should first orient the participant to the treadmill showing them how to stop it if necessary. The participant should wear the emergency shut off band. Demonstrate what is meant by a good heel strike. If you notice 3 consecutive steps in which the feet scuff the treadmill, provide verbal cue. Place a gait belt on the participant. Stand behind the participant on the treadmill platform with your hands near the participant’s waist to guard for safety.

**Instructions:** I will ask you to talk on the treadmill for 10 minutes at a pace that is slightly above your comfortable pace. If at any point you feel you are short of breath or are unable to continue, please tell the physical therapist or push the red stop button. Please focus on taking consistent strides with a good heel strike at each step. You may hold onto the railings if this helps you feel more stable.

**Treadmill Walking Pace:**

**10% Above Comfortable Overground Pace (take from Week 8, Visit 1): ___________________mph**

**Verbal Cues for Feedback:**

- Continue focusing on good heel strike with each step.
- Try to stand up straight as you walk.

**Successful Repetition:**

- Completes 10 minutes of treadmill walking with no more than 10 verbal cues.

**Unsuccessful Repetition:**

- Unable to complete 10 minutes of treadmill walking.
- Requires more than 10 verbal cues over the 10 minutes.

**Did you have to adjust the pace of the treadmill? YES / NO**

**If YES, at which time point did you adjust the pace? ________________**

**If YES, to which speed did you set the treadmill? ________________mph.**

**If YES, was the participant able to finish the treadmill session at this pace? YES / NO**

**Stability in Gait**

**Dual Task Walking**

**WEEK 1, VISIT 1:** Walking with MOTOR dual task

**Equipment:** None

Instructions: I want you to walk overground at your comfortable pace while bouncing this ball. While bouncing the ball, continue to focus on walking with a good stride and heel strike. You will perform 6 trials.

**Verbal Cues for Feedback:**

- Try to maintain control of the ball as you bounce it.
- Continue walking with a good stride and heel strike.

**Successful Repetition:**

- Does not lose balance or stumble while walking.
- Completes at least 4 bounces for each trial.

**Unsuccessful Repetition:**

- Loses balance or stumbles.
- Completes less than or equal to 3 bounces for each trial.

**Performance:**

- **Trial 1:** _____________bounces Maintain good balance: YES / NO
- **Trial 2:** _____________bounces Maintain good balance: YES / NO
- **Trial 3:** ____________­_bounces Maintain good balance: YES / NO
- **Trial 4:** _____________bounces Maintain good balance: YES / NO
- **Trial 5:** _____________bounces Maintain good balance: YES / NO
- **Trial 6:** _____________bounces Maintain good balance: YES / NO

**Stability in Gait**

**Dual Task Walking**

**WEEK 1, VISIT 2:** Walking with MOTOR dual task

**Equipment:** None

Instructions: I want you to walk overground at your comfortable pace while bouncing this ball. While bouncing the ball, continue to focus on walking with a good stride and heel strike. You will perform 6 trials.

**Verbal Cues for Feedback:**

- Try to maintain control of the ball as you bounce it.
- Continue walking with a good stride and heel strike.

**Successful Repetition:**

- Does not lose balance or stumble while walking.
- Completes at least 4 bounces for each trial.

**Unsuccessful Repetition:**

- Loses balance or stumbles.
- Completes less than or equal to 3 bounces for each trial.

**Performance:**

- **Trial 1:** _____________bounces Maintain good balance: YES / NO
- **Trial 2:** _____________bounces Maintain good balance: YES / NO
- **Trial 3:** ____________­_bounces Maintain good balance: YES / NO
- **Trial 4:** _____________bounces Maintain good balance: YES / NO
- **Trial 5:** _____________bounces Maintain good balance: YES / NO
- **Trial 6:** _____________bounces Maintain good balance: YES / NO

**Stability in Gait**

**Dual Task Walking**

**WEEK 2, VISIT 1:** Walking with MOTOR dual task

**Equipment:** None

Instructions: I want you to walk overground at your comfortable pace while bouncing this ball. While bouncing the ball, continue to focus on walking with a good stride and heel strike. You will perform 6 trials.

**Verbal Cues for Feedback:**

- Try to maintain control of the ball as you bounce it.
- Continue walking with a good stride and heel strike.

**Successful Repetition:**

- Does not lose balance or stumble while walking.
- Completes at least 4 bounces for each trial.

**Unsuccessful Repetition:**

- Loses balance or stumbles.
- Completes less than or equal to 3 bounces for each trial.

**Performance:**

- **Trial 1:** _____________bounces Maintain good balance: YES / NO
- **Trial 2:** _____________bounces Maintain good balance: YES / NO
- **Trial 3:** ____________­_bounces Maintain good balance: YES / NO
- **Trial 4:** _____________bounces Maintain good balance: YES / NO
- **Trial 5:** _____________bounces Maintain good balance: YES / NO
- **Trial 6:** _____________bounces Maintain good balance: YES / NO

**Stability in Gait**

**Dual Task Walking**

**WEEK 2, VISIT 2:** Walking with MOTOR dual task

**Equipment:** None

Instructions: I want you to walk overground at your comfortable pace while bouncing this ball. While bouncing the ball, continue to focus on walking with a good stride and heel strike. You will perform 6 trials.

**Verbal Cues for Feedback:**

- Try to maintain control of the ball as you bounce it.
- Continue walking with a good stride and heel strike.

**Successful Repetition:**

- Does not lose balance or stumble while walking.
- Completes at least 4 bounces for each trial.

**Unsuccessful Repetition:**

- Loses balance or stumbles.
- Completes less than or equal to 3 bounces for each trial.

**Performance:**

- **Trial 1:** _____________bounces Maintain good balance: YES / NO
- **Trial 2:** _____________bounces Maintain good balance: YES / NO
- **Trial 3:** ____________­_bounces Maintain good balance: YES / NO
- **Trial 4:** _____________bounces Maintain good balance: YES / NO
- **Trial 5:** _____________bounces Maintain good balance: YES / NO
- **Trial 6:** _____________bounces Maintain good balance: YES / NO

**Stability in Gait**

**Dual Task Walking**

**WEEK 3, VISIT 1:** Walking with MOTOR dual task

**Equipment:** None

Instructions: I want you to walk overground at your comfortable pace while bouncing this ball. While bouncing the ball, continue to focus on walking with a good stride and heel strike. You will perform 6 trials.

**Verbal Cues for Feedback:**

- Try to maintain control of the ball as you bounce it.
- Continue walking with a good stride and heel strike.

**Successful Repetition:**

- Does not lose balance or stumble while walking.
- Completes at least 4 bounces for each trial.

**Unsuccessful Repetition:**

- Loses balance or stumbles.
- Completes less than or equal to 3 bounces for each trial.

**Performance:**

- **Trial 1:** _____________bounces Maintain good balance: YES / NO
- **Trial 2:** _____________bounces Maintain good balance: YES / NO
- **Trial 3:** ____________­_bounces Maintain good balance: YES / NO
- **Trial 4:** _____________bounces Maintain good balance: YES / NO
- **Trial 5:** _____________bounces Maintain good balance: YES / NO
- **Trial 6:** _____________bounces Maintain good balance: YES / NO

**Stability in Gait**

**Dual Task Walking**

**WEEK 3, VISIT 2:** Walking with MOTOR dual task

**Equipment:** None

Instructions: I want you to walk overground at your comfortable pace while bouncing this ball. While bouncing the ball, continue to focus on walking with a good stride and heel strike. You will perform 6 trials.

**Verbal Cues for Feedback:**

- Try to maintain control of the ball as you bounce it.
- Continue walking with a good stride and heel strike.

**Successful Repetition:**

- Does not lose balance or stumble while walking.
- Completes at least 4 bounces for each trial.

**Unsuccessful Repetition:**

- Loses balance or stumbles.
- Completes less than or equal to 3 bounces for each trial.

**Performance:**

- **Trial 1:** _____________bounces Maintain good balance: YES / NO
- **Trial 2:** _____________bounces Maintain good balance: YES / NO
- **Trial 3:** ____________­_bounces Maintain good balance: YES / NO
- **Trial 4:** _____________bounces Maintain good balance: YES / NO
- **Trial 5:** _____________bounces Maintain good balance: YES / NO
- **Trial 6:** _____________bounces Maintain good balance: YES / NO

**Stability in Gait**

**Dual Task Walking**

**WEEK 4, VISIT 1:** Walking with MOTOR dual task

**Equipment:** None

Instructions: I want you to walk overground at your comfortable pace while bouncing this ball. While bouncing the ball, continue to focus on walking with a good stride and heel strike. You will perform 6 trials.

**Verbal Cues for Feedback:**

- Try to maintain control of the ball as you bounce it.
- Continue walking with a good stride and heel strike.

**Successful Repetition:**

- Does not lose balance or stumble while walking.
- Completes at least 4 bounces for each trial.

**Unsuccessful Repetition:**

- Loses balance or stumbles.
- Completes less than or equal to 3 bounces for each trial.

**Performance:**

- **Trial 1:** _____________bounces Maintain good balance: YES / NO
- **Trial 2:** _____________bounces Maintain good balance: YES / NO
- **Trial 3:** ____________­_bounces Maintain good balance: YES / NO
- **Trial 4:** _____________bounces Maintain good balance: YES / NO
- **Trial 5:** _____________bounces Maintain good balance: YES / NO
- **Trial 6:** _____________bounces Maintain good balance: YES / NO

**Stability in Gait**

**Dual Task Walking**

**WEEK 4, VISIT 2:** Walking with MOTOR dual task

**Equipment:** None

Instructions: I want you to walk overground at your comfortable pace while bouncing this ball. While bouncing the ball, continue to focus on walking with a good stride and heel strike. You will perform 6 trials.

**Verbal Cues for Feedback:**

- Try to maintain control of the ball as you bounce it.
- Continue walking with a good stride and heel strike.

**Successful Repetition:**

- Does not lose balance or stumble while walking.
- Completes at least 4 bounces for each trial.

**Unsuccessful Repetition:**

- Loses balance or stumbles.
- Completes less than or equal to 3 bounces for each trial.

**Performance:**

- **Trial 1:** _____________bounces Maintain good balance: YES / NO
- **Trial 2:** _____________bounces Maintain good balance: YES / NO
- **Trial 3:** ____________­_bounces Maintain good balance: YES / NO
- **Trial 4:** _____________bounces Maintain good balance: YES / NO
- **Trial 5:** _____________bounces Maintain good balance: YES / NO
- **Trial 6:** _____________bounces Maintain good balance: YES / NO

**Stability in Gait**

**Dual Task Walking**

**WEEK 5, VISIT 1:** Walking with MOTOR dual task

**Equipment:** None

Instructions: I want you to walk overground at your comfortable pace while bouncing this ball. While bouncing the ball, continue to focus on walking with a good stride and heel strike. You will perform 6 trials.

**Verbal Cues for Feedback:**

- Try to maintain control of the ball as you bounce it.
- Continue walking with a good stride and heel strike.

**Successful Repetition:**

- Does not lose balance or stumble while walking.
- Completes at least 4 bounces for each trial.

**Unsuccessful Repetition:**

- Loses balance or stumbles.
- Completes less than or equal to 3 bounces for each trial.

**Performance:**

- **Trial 1:** _____________bounces Maintain good balance: YES / NO
- **Trial 2:** _____________bounces Maintain good balance: YES / NO
- **Trial 3:** ____________­_bounces Maintain good balance: YES / NO
- **Trial 4:** _____________bounces Maintain good balance: YES / NO
- **Trial 5:** _____________bounces Maintain good balance: YES / NO
- **Trial 6:** _____________bounces Maintain good balance: YES / NO

**Stability in Gait**

**Dual Task Walking**

**WEEK 5, VISIT 2:** Walking with MOTOR dual task

**Equipment:** None

Instructions: I want you to walk overground at your comfortable pace while bouncing this ball. While bouncing the ball, continue to focus on walking with a good stride and heel strike. You will perform 6 trials.

**Verbal Cues for Feedback:**

- Try to maintain control of the ball as you bounce it.
- Continue walking with a good stride and heel strike.

**Successful Repetition:**

- Does not lose balance or stumble while walking.
- Completes at least 4 bounces for each trial.

**Unsuccessful Repetition:**

- Loses balance or stumbles.
- Completes less than or equal to 3 bounces for each trial.

**Performance:**

- **Trial 1:** _____________bounces Maintain good balance: YES / NO
- **Trial 2:** _____________bounces Maintain good balance: YES / NO
- **Trial 3:** ____________­_bounces Maintain good balance: YES / NO
- **Trial 4:** _____________bounces Maintain good balance: YES / NO
- **Trial 5:** _____________bounces Maintain good balance: YES / NO
- **Trial 6:** _____________bounces Maintain good balance: YES / NO

**Stability in Gait**

**Dual Task Walking**

**WEEK 6, VISIT 1:** Walking with MOTOR dual task

**Equipment:** None

Instructions: I want you to walk overground at your comfortable pace while bouncing this ball. While bouncing the ball, continue to focus on walking with a good stride and heel strike. You will perform 6 trials.

**Verbal Cues for Feedback:**

- Try to maintain control of the ball as you bounce it.
- Continue walking with a good stride and heel strike.

**Successful Repetition:**

- Does not lose balance or stumble while walking.
- Completes at least 4 bounces for each trial.

**Unsuccessful Repetition:**

- Loses balance or stumbles.
- Completes less than or equal to 3 bounces for each trial.

**Performance:**

- **Trial 1:** _____________bounces Maintain good balance: YES / NO
- **Trial 2:** _____________bounces Maintain good balance: YES / NO
- **Trial 3:** ____________­_bounces Maintain good balance: YES / NO
- **Trial 4:** _____________bounces Maintain good balance: YES / NO
- **Trial 5:** _____________bounces Maintain good balance: YES / NO
- **Trial 6:** _____________bounces Maintain good balance: YES / NO

**Stability in Gait**

**Dual Task Walking**

**WEEK 6, VISIT 2:** Walking with MOTOR dual task

**Equipment:** None

Instructions: I want you to walk overground at your comfortable pace while bouncing this ball. While bouncing the ball, continue to focus on walking with a good stride and heel strike. You will perform 6 trials.

**Verbal Cues for Feedback:**

- Try to maintain control of the ball as you bounce it.
- Continue walking with a good stride and heel strike.

**Successful Repetition:**

- Does not lose balance or stumble while walking.
- Completes at least 4 bounces for each trial.

**Unsuccessful Repetition:**

- Loses balance or stumbles.
- Completes less than or equal to 3 bounces for each trial.

**Performance:**

- **Trial 1:** _____________bounces Maintain good balance: YES / NO
- **Trial 2:** _____________bounces Maintain good balance: YES / NO
- **Trial 3:** ____________­_bounces Maintain good balance: YES / NO
- **Trial 4:** _____________bounces Maintain good balance: YES / NO
- **Trial 5:** _____________bounces Maintain good balance: YES / NO
- **Trial 6:** _____________bounces Maintain good balance: YES / NO

**Stability in Gait**

**Dual Task Walking**

**WEEK 7, VISIT 1:** Walking with MOTOR dual task

**Equipment:** None

Instructions: I want you to walk overground at your comfortable pace while bouncing this ball. While bouncing the ball, continue to focus on walking with a good stride and heel strike. You will perform 6 trials.

**Verbal Cues for Feedback:**

- Try to maintain control of the ball as you bounce it.
- Continue walking with a good stride and heel strike.

**Successful Repetition:**

- Does not lose balance or stumble while walking.
- Completes at least 4 bounces for each trial.

**Unsuccessful Repetition:**

- Loses balance or stumbles.
- Completes less than or equal to 3 bounces for each trial.

**Performance:**

- **Trial 1:** _____________bounces Maintain good balance: YES / NO
- **Trial 2:** _____________bounces Maintain good balance: YES / NO
- **Trial 3:** ____________­_bounces Maintain good balance: YES / NO
- **Trial 4:** _____________bounces Maintain good balance: YES / NO
- **Trial 5:** _____________bounces Maintain good balance: YES / NO
- **Trial 6:** _____________bounces Maintain good balance: YES / NO

**Stability in Gait**

**Dual Task Walking**

**WEEK 7, VISIT 2:** Walking with MOTOR dual task

**Equipment:** None

Instructions: I want you to walk overground at your comfortable pace while bouncing this ball. While bouncing the ball, continue to focus on walking with a good stride and heel strike. You will perform 6 trials.

**Verbal Cues for Feedback:**

- Try to maintain control of the ball as you bounce it.
- Continue walking with a good stride and heel strike.

**Successful Repetition:**

- Does not lose balance or stumble while walking.
- Completes at least 4 bounces for each trial.

**Unsuccessful Repetition:**

- Loses balance or stumbles.
- Completes less than or equal to 3 bounces for each trial.

**Performance:**

- **Trial 1:** _____________bounces Maintain good balance: YES / NO
- **Trial 2:** _____________bounces Maintain good balance: YES / NO
- **Trial 3:** ____________­_bounces Maintain good balance: YES / NO
- **Trial 4:** _____________bounces Maintain good balance: YES / NO
- **Trial 5:** _____________bounces Maintain good balance: YES / NO
- **Trial 6:** _____________bounces Maintain good balance: YES / NO

**Stability in Gait**

**Dual Task Walking**

**WEEK 8, VISIT 1:** Walking with MOTOR dual task

**Equipment:** None

Instructions: I want you to walk overground at your comfortable pace while bouncing this ball. While bouncing the ball, continue to focus on walking with a good stride and heel strike. You will perform 6 trials.

**Verbal Cues for Feedback:**

- Try to maintain control of the ball as you bounce it.
- Continue walking with a good stride and heel strike.

**Successful Repetition:**

- Does not lose balance or stumble while walking.
- Completes at least 4 bounces for each trial.

**Unsuccessful Repetition:**

- Loses balance or stumbles.
- Completes less than or equal to 3 bounces for each trial.

**Performance:**

- **Trial 1:** _____________bounces Maintain good balance: YES / NO
- **Trial 2:** _____________bounces Maintain good balance: YES / NO
- **Trial 3:** ____________­_bounces Maintain good balance: YES / NO
- **Trial 4:** _____________bounces Maintain good balance: YES / NO
- **Trial 5:** _____________bounces Maintain good balance: YES / NO
- **Trial 6:** _____________bounces Maintain good balance: YES / NO

**Stability in Gait**

**Dual Task Walking**

**WEEK 8, VISIT 2:** Walking with MOTOR dual task

**Equipment:** None

Instructions: I want you to walk overground at your comfortable pace while bouncing this ball. While bouncing the ball, continue to focus on walking with a good stride and heel strike. You will perform 6 trials.

**Verbal Cues for Feedback:**

- Try to maintain control of the ball as you bounce it.
- Continue walking with a good stride and heel strike.

**Successful Repetition:**

- Does not lose balance or stumble while walking.
- Completes at least 4 bounces for each trial.

**Unsuccessful Repetition:**

- Loses balance or stumbles.
- Completes less than or equal to 3 bounces for each trial.

**Performance:**

- **Trial 1:** _____________bounces Maintain good balance: YES / NO
- **Trial 2:** _____________bounces Maintain good balance: YES / NO
- **Trial 3:** ____________­_bounces Maintain good balance: YES / NO
- **Trial 4:** _____________bounces Maintain good balance: YES / NO
- **Trial 5:** _____________bounces Maintain good balance: YES / NO
- **Trial 6:** _____________bounces Maintain good balance: YES / NO

**Stability in Gait**

**Dual Task Walking**

**WEEK 1, VISIT 1:** Walking with COGNITIVE dual task

**Equipment:** None

Instructions: I want you to walk overground at your comfortable pace while naming items that fall into a certain category, which I will provide to you. While naming the items, continue to focus on walking with a good stride and heel strike. You will perform 6 trials.

- Category 1: Objects in the sky
- Category 2: Things that are RED
- Category 3: Games you play with a ball
- Category 4: Words that begin with “R”
- Category 5: Foods
- Category 6: State names within the United States

**Verbal Cues for Feedback:**

- Continue walking with a good stride and heel strike.
- Continue naming items in the category.

**Successful Repetition:**

- Does not lose balance or stumble while walking.
- Names at least 2 items per category for each trial.

**Unsuccessful Repetition:**

- Loses balance or stumbles.
- Names less than or equal to 1 item per category for each trial.

**Performance:**

- **Trial 1:** _____________items listed Maintain good balance: YES / NO
- **Trial 2:** _____________items listed Maintain good balance: YES / NO
- **Trial 3:** _____________items listed Maintain good balance: YES / NO
- **Trial 4:** _____________items listed Maintain good balance: YES / NO
- **Trial 5:** _____________items listed Maintain good balance: YES / NO
- **Trial 6:** _____________items listed Maintain good balance: YES / NO

**Stability in Gait**

**Dual Task Walking**

**WEEK 1, VISIT 2:** Walking with COGNITIVE dual task

**Equipment:** None

Instructions: I want you to walk overground at your comfortable pace while naming items that fall into a certain category, which I will provide to you. While naming the items, continue to focus on walking with a good stride and heel strike. You will perform 6 trials.

- Category 1: Makes of cars
- Category 2: Things that are BLUE
- Category 3: Types of Candy
- Category 4: Words that begin with “M”
- Category 5: Beverages
- Category 6: Countries

**Verbal Cues for Feedback:**

- Continue walking with a good stride and heel strike.
- Continue naming items in the category.

**Successful Repetition:**

- Does not lose balance or stumble while walking.
- Names at least 2 items per category for each trial.

**Unsuccessful Repetition:**

- Loses balance or stumbles.
- Names less than or equal to 1 item per category for each trial.

**Performance:**

- **Trial 1:** _____________items listed Maintain good balance: YES / NO
- **Trial 2:** _____________items listed Maintain good balance: YES / NO
- **Trial 3:** _____________items listed Maintain good balance: YES / NO
- **Trial 4:** _____________items listed Maintain good balance: YES / NO
- **Trial 5:** _____________items listed Maintain good balance: YES / NO
- **Trial 6:** _____________items listed Maintain good balance: YES / NO

**Stability in Gait**

**Dual Task Walking**

**WEEK 2, VISIT 1:** Walking with COGNITIVE dual task

**Equipment:** None

Instructions: I want you to walk overground at your comfortable pace while naming items that fall into a certain category, which I will provide to you. While naming the items, continue to focus on walking with a good stride and heel strike. You will perform 6 trials.

- Category 1: Restaurants
- Category 2: Things that are GREEN
- Category 3: Board Games
- Category 4: Words that begin with “B”
- Category 5: Desserts
- Category 6: Cities in the United States

**Verbal Cues for Feedback:**

- Continue walking with a good stride and heel strike.
- Continue naming items in the category.

**Successful Repetition:**

- Does not lose balance or stumble while walking.
- Names at least 2 items per category for each trial.

**Unsuccessful Repetition:**

- Loses balance or stumbles.
- Names less than or equal to 1 item per category for each trial.

**Performance:**

- **Trial 1:** _____________items listed Maintain good balance: YES / NO
- **Trial 2:** _____________items listed Maintain good balance: YES / NO
- **Trial 3:** _____________items listed Maintain good balance: YES / NO
- **Trial 4:** _____________items listed Maintain good balance: YES / NO
- **Trial 5:** _____________items listed Maintain good balance: YES / NO
- **Trial 6:** _____________items listed Maintain good balance: YES / NO

**Stability in Gait**

**Dual Task Walking**

**WEEK 2, VISIT 2:** Walking with COGNITIVE dual task

**Equipment:** None

Instructions: I want you to walk overground at your comfortable pace while naming items that fall into a certain category, which I will provide to you. While naming the items, continue to focus on walking with a good stride and heel strike. You will perform 6 trials.

- Category 1: Animals
- Category 2: Things that are YELLOW
- Category 3: Fruits
- Category 4: Words that begin with “W”
- Category 5: Holidays
- Category 6: Famous Persons

**Verbal Cues for Feedback:**

- Continue walking with a good stride and heel strike.
- Continue naming items in the category.

**Successful Repetition:**

- Does not lose balance or stumble while walking.
- Names at least 2 items per category for each trial.

**Unsuccessful Repetition:**

- Loses balance or stumbles.
- Names less than or equal to 1 item per category for each trial.

**Performance:**

- **Trial 1:** _____________items listed Maintain good balance: YES / NO
- **Trial 2:** _____________items listed Maintain good balance: YES / NO
- **Trial 3:** _____________items listed Maintain good balance: YES / NO
- **Trial 4:** _____________items listed Maintain good balance: YES / NO
- **Trial 5:** _____________items listed Maintain good balance: YES / NO
- **Trial 6:** _____________items listed Maintain good balance: YES / NO

**Stability in Gait**

**Dual Task Walking**

**WEEK 3, VISIT 1:** Walking with COGNITIVE dual task

**Equipment:** None

Instructions: I want you to walk overground at your comfortable pace while naming items that fall into a certain category, which I will provide to you. While naming the items, continue to focus on walking with a good stride and heel strike. You will perform 6 trials.

- Category 1: Modes of transportation
- Category 2: Things that are ORANGE
- Category 3: Office materials/equipment
- Category 4: Words that begin with “N”
- Category 5: Breakfast foods
- Category 6: Vacation/holiday destinations

**Verbal Cues for Feedback:**

- Continue walking with a good stride and heel strike.
- Continue naming items in the category.

**Successful Repetition:**

- Does not lose balance or stumble while walking.
- Names at least 2 items per category for each trial.

**Unsuccessful Repetition:**

- Loses balance or stumbles.
- Names less than or equal to 1 item per category for each trial.

**Performance:**

- **Trial 1:** _____________items listed Maintain good balance: YES / NO
- **Trial 2:** _____________items listed Maintain good balance: YES / NO
- **Trial 3:** _____________items listed Maintain good balance: YES / NO
- **Trial 4:** _____________items listed Maintain good balance: YES / NO
- **Trial 5:** _____________items listed Maintain good balance: YES / NO
- **Trial 6:** _____________items listed Maintain good balance: YES / NO

**Stability in Gait**

**Dual Task Walking**

**WEEK 3, VISIT 2:** Walking with COGNITIVE dual task

**Equipment:** None

Instructions: I want you to walk overground at your comfortable pace while naming items that fall into a certain category, which I will provide to you. While naming the items, continue to focus on walking with a good stride and heel strike. You will perform 6 trials.

- Category 1: Clothing items
- Category 2: Vegetables
- Category 3: Names of girls
- Category 4: Words that begin with “J”
- Category 5: Sports
- Category 6: Pieces of furniture

**Verbal Cues for Feedback:**

- Continue walking with a good stride and heel strike.
- Continue naming items in the category.

**Successful Repetition:**

- Does not lose balance or stumble while walking.
- Names at least 2 items per category for each trial.

**Unsuccessful Repetition:**

- Loses balance or stumbles.
- Names less than or equal to 1 item per category for each trial.

**Performance:**

- **Trial 1:** _____________items listed Maintain good balance: YES / NO
- **Trial 2:** _____________items listed Maintain good balance: YES / NO
- **Trial 3:** _____________items listed Maintain good balance: YES / NO
- **Trial 4:** _____________items listed Maintain good balance: YES / NO
- **Trial 5:** _____________items listed Maintain good balance: YES / NO
- **Trial 6:** _____________items listed Maintain good balance: YES / NO

**Stability in Gait**

**Dual Task Walking**

**WEEK 4, VISIT 1:** Walking with COGNITIVE dual task

**Equipment:** None

Instructions: I want you to walk overground at your comfortable pace while naming items that fall into a certain category, which I will provide to you. While naming the items, continue to focus on walking with a good stride and heel strike. You will perform 6 trials.

- Category 1: Games you play with a ball
- Category 2: State names within the United States
- Category 3: Objects in the sky
- Category 4: Foods
- Category 5: Words that begin with “R”
- Category 6: Things that are RED

**Verbal Cues for Feedback:**

- Continue walking with a good stride and heel strike.
- Continue naming items in the category.

**Successful Repetition:**

- Does not lose balance or stumble while walking.
- Names at least 2 items per category for each trial.

**Unsuccessful Repetition:**

- Loses balance or stumbles.
- Names less than or equal to 1 item per category for each trial.

**Performance:**

- **Trial 1:** _____________items listed Maintain good balance: YES / NO
- **Trial 2:** _____________items listed Maintain good balance: YES / NO
- **Trial 3:** _____________items listed Maintain good balance: YES / NO
- **Trial 4:** _____________items listed Maintain good balance: YES / NO
- **Trial 5:** _____________items listed Maintain good balance: YES / NO
- **Trial 6:** _____________items listed Maintain good balance: YES / NO

**Stability in Gait**

**Dual Task Walking**

**WEEK 4, VISIT 2:** Walking with COGNITIVE dual task

**Equipment:** None

Instructions: I want you to walk overground at your comfortable pace while naming items that fall into a certain category, which I will provide to you. While naming the items, continue to focus on walking with a good stride and heel strike. You will perform 6 trials.

- Category 1: Beverages
- Category 2: Types of Candy
- Category 3: Things that are BLUE
- Category 4: Countries
- Category 5: Makes of cars
- Category 6: Words that begin with “M”

**Verbal Cues for Feedback:**

- Continue walking with a good stride and heel strike.
- Continue naming items in the category.

**Successful Repetition:**

- Does not lose balance or stumble while walking.
- Names at least 2 items per category for each trial.

**Unsuccessful Repetition:**

- Loses balance or stumbles.
- Names less than or equal to 1 item per category for each trial.

**Performance:**

- **Trial 1:** _____________items listed Maintain good balance: YES / NO
- **Trial 2:** _____________items listed Maintain good balance: YES / NO
- **Trial 3:** _____________items listed Maintain good balance: YES / NO
- **Trial 4:** _____________items listed Maintain good balance: YES / NO
- **Trial 5:** _____________items listed Maintain good balance: YES / NO
- **Trial 6:** _____________items listed Maintain good balance: YES / NO

**Stability in Gait**

**Dual Task Walking**

**WEEK 5, VISIT 1:** Walking with COGNITIVE dual task

**Equipment:** None

Instructions: I want you to walk overground at your comfortable pace while naming items that fall into a certain category, which I will provide to you. While naming the items, continue to focus on walking with a good stride and heel strike. You will perform 6 trials.

- Category 1: Things that are GREEN
- Category 2: Restaurants
- Category 3: Cities in the United States
- Category 4: Desserts
- Category 5: Words that begin with “B”
- Category 6: Board Games

**Verbal Cues for Feedback:**

- Continue walking with a good stride and heel strike.
- Continue naming items in the category.

**Successful Repetition:**

- Does not lose balance or stumble while walking.
- Names at least 2 items per category for each trial.

**Unsuccessful Repetition:**

- Loses balance or stumbles.
- Names less than or equal to 1 item per category for each trial.

**Performance:**

- **Trial 1:** _____________items listed Maintain good balance: YES / NO
- **Trial 2:** _____________items listed Maintain good balance: YES / NO
- **Trial 3:** _____________items listed Maintain good balance: YES / NO
- **Trial 4:** _____________items listed Maintain good balance: YES / NO
- **Trial 5:** _____________items listed Maintain good balance: YES / NO
- **Trial 6:** _____________items listed Maintain good balance: YES / NO

**Stability in Gait**

**Dual Task Walking**

**WEEK 5, VISIT 2:** Walking with COGNITIVE dual task

**Equipment:** None

Instructions: I want you to walk overground at your comfortable pace while naming items that fall into a certain category, which I will provide to you. While naming the items, continue to focus on walking with a good stride and heel strike. You will perform 6 trials.

- Category 1: Holidays Animals
- Category 2: Words that begin with “W”
- Category 3: Famous Persons
- Category 4: Things that are YELLOW
- Category 5: Animals
- Category 6: Fruits Famous Persons

**Verbal Cues for Feedback:**

- Continue walking with a good stride and heel strike.
- Continue naming items in the category.

**Successful Repetition:**

- Does not lose balance or stumble while walking.
- Names at least 2 items per category for each trial.

**Unsuccessful Repetition:**

- Loses balance or stumbles.
- Names less than or equal to 1 item per category for each trial.

**Performance:**

- **Trial 1:** _____________items listed Maintain good balance: YES / NO
- **Trial 2:** _____________items listed Maintain good balance: YES / NO
- **Trial 3:** _____________items listed Maintain good balance: YES / NO
- **Trial 4:** _____________items listed Maintain good balance: YES / NO
- **Trial 5:** _____________items listed Maintain good balance: YES / NO
- **Trial 6:** _____________items listed Maintain good balance: YES / NO

**Stability in Gait**

**Dual Task Walking**

**WEEK 6, VISIT 1:** Walking with COGNITIVE dual task

**Equipment:** None

Instructions: I want you to walk overground at your comfortable pace while naming items that fall into a certain category, which I will provide to you. While naming the items, continue to focus on walking with a good stride and heel strike. You will perform 6 trials.

- Category 1: Words that begin with “N”
- Category 2: Vacation/holiday destinations
- Category 3: Breakfast foods
- Category 4: Things that are ORANGE
- Category 5: Office materials/equipment
- Category 6: Modes of transportation

**Verbal Cues for Feedback:**

- Continue walking with a good stride and heel strike.
- Continue naming items in the category.

**Successful Repetition:**

- Does not lose balance or stumble while walking.
- Names at least 2 items per category for each trial.

**Unsuccessful Repetition:**

- Loses balance or stumbles.
- Names less than or equal to 1 item per category for each trial.

**Performance:**

- **Trial 1:** _____________items listed Maintain good balance: YES / NO
- **Trial 2:** _____________items listed Maintain good balance: YES / NO
- **Trial 3:** _____________items listed Maintain good balance: YES / NO
- **Trial 4:** _____________items listed Maintain good balance: YES / NO
- **Trial 5:** _____________items listed Maintain good balance: YES / NO
- **Trial 6:** _____________items listed Maintain good balance: YES / NO

**Stability in Gait**

**Dual Task Walking**

**WEEK 6, VISIT 2:** Walking with COGNITIVE dual task

**Equipment:** None

Instructions: I want you to walk overground at your comfortable pace while naming items that fall into a certain category, which I will provide to you. While naming the items, continue to focus on walking with a good stride and heel strike. You will perform 6 trials.

- Category 1: Sports
- Category 2: Pieces of furniture
- Category 3: Words that begin with “J”
- Category 4: Names of girls
- Category 5: Clothing items
- Category 6: Vegetables

**Verbal Cues for Feedback:**

- Continue walking with a good stride and heel strike.
- Continue naming items in the category.

**Successful Repetition:**

- Does not lose balance or stumble while walking.
- Names at least 2 items per category for each trial.

**Unsuccessful Repetition:**

- Loses balance or stumbles.
- Names less than or equal to 1 item per category for each trial.

**Performance:**

- **Trial 1:** _____________items listed Maintain good balance: YES / NO
- **Trial 2:** _____________items listed Maintain good balance: YES / NO
- **Trial 3:** _____________items listed Maintain good balance: YES / NO
- **Trial 4:** _____________items listed Maintain good balance: YES / NO
- **Trial 5:** _____________items listed Maintain good balance: YES / NO
- **Trial 6:** _____________items listed Maintain good balance: YES / NO

**Stability in Gait**

**Dual Task Walking**

**WEEK 7, VISIT 1:** Walking with COGNITIVE dual task

**Equipment:** None

Instructions: I want you to walk overground at your comfortable pace while naming items that fall into a certain category, which I will provide to you. While naming the items, continue to focus on walking with a good stride and heel strike. You will perform 6 trials.

- Category 1: Games you play with a ball
- Category 2: State names within the United States
- Category 3: Objects in the sky
- Category 4: Foods
- Category 5: Words that begin with “R”
- Category 6: Things that are RED

**Verbal Cues for Feedback:**

- Continue walking with a good stride and heel strike.
- Continue naming items in the category.

**Successful Repetition:**

- Does not lose balance or stumble while walking.
- Names at least 2 items per category for each trial.

**Unsuccessful Repetition:**

- Loses balance or stumbles.
- Names less than or equal to 1 item per category for each trial.

**Performance:**

- **Trial 1:** _____________items listed Maintain good balance: YES / NO
- **Trial 2:** _____________items listed Maintain good balance: YES / NO
- **Trial 3:** _____________items listed Maintain good balance: YES / NO
- **Trial 4:** _____________items listed Maintain good balance: YES / NO
- **Trial 5:** _____________items listed Maintain good balance: YES / NO
- **Trial 6:** _____________items listed Maintain good balance: YES / NO

**Stability in Gait**

**Dual Task Walking**

**WEEK 7, VISIT 2:** Walking with COGNITIVE dual task

**Equipment:** None

Instructions: I want you to walk overground at your comfortable pace while naming items that fall into a certain category, which I will provide to you. While naming the items, continue to focus on walking with a good stride and heel strike. You will perform 6 trials.

- Category 1: Words that begin with “M”
- Category 2: Beverages
- Category 3: Countries
- Category 4: Types of Candy
- Category 5: Things that are BLUE
- Category 6: Makes of cars

**Verbal Cues for Feedback:**

- Continue walking with a good stride and heel strike.
- Continue naming items in the category.

**Successful Repetition:**

- Does not lose balance or stumble while walking.
- Names at least 2 items per category for each trial.

**Unsuccessful Repetition:**

- Loses balance or stumbles.
- Names less than or equal to 1 item per category for each trial.

**Performance:**

- **Trial 1:** _____________items listed Maintain good balance: YES / NO
- **Trial 2:** _____________items listed Maintain good balance: YES / NO
- **Trial 3:** _____________items listed Maintain good balance: YES / NO
- **Trial 4:** _____________items listed Maintain good balance: YES / NO
- **Trial 5:** _____________items listed Maintain good balance: YES / NO
- **Trial 6:** _____________items listed Maintain good balance: YES / NO

**Stability in Gait**

**Dual Task Walking**

**WEEK 8, VISIT 1:** Walking with COGNITIVE dual task

**Equipment:** None

Instructions: I want you to walk overground at your comfortable pace while naming items that fall into a certain category, which I will provide to you. While naming the items, continue to focus on walking with a good stride and heel strike. You will perform 6 trials.

- Category 1: Cities in the United States
- Category 2: Desserts
- Category 3: Words that begin with “B”
- Category 4: Board Games
- Category 5: Things that are GREEN
- Category 6: Restaurants

**Verbal Cues for Feedback:**

- Continue walking with a good stride and heel strike.
- Continue naming items in the category.

**Successful Repetition:**

- Does not lose balance or stumble while walking.
- Names at least 2 items per category for each trial.

**Unsuccessful Repetition:**

- Loses balance or stumbles.
- Names less than or equal to 1 item per category for each trial.

**Performance:**

- **Trial 1:** _____________items listed Maintain good balance: YES / NO
- **Trial 2:** _____________items listed Maintain good balance: YES / NO
- **Trial 3:** _____________items listed Maintain good balance: YES / NO
- **Trial 4:** _____________items listed Maintain good balance: YES / NO
- **Trial 5:** _____________items listed Maintain good balance: YES / NO
- **Trial 6:** _____________items listed Maintain good balance: YES / NO

**Stability in Gait**

**Dual Task Walking**

**WEEK 8, VISIT 2:** Walking with COGNITIVE dual task

**Equipment:** None

Instructions: I want you to walk overground at your comfortable pace while naming items that fall into a certain category, which I will provide to you. While naming the items, continue to focus on walking with a good stride and heel strike. You will perform 6 trials.

- Category 1: Things that are YELLOW
- Category 2: Holidays
- Category 3: Words that begin with “W”
- Category 4: Fruits
- Category 5: Famous Persons
- Category 6: Animals

**Verbal Cues for Feedback:**

- Continue walking with a good stride and heel strike.
- Continue naming items in the category.

**Successful Repetition:**

- Does not lose balance or stumble while walking.
- Names at least 2 items per category for each trial.

**Unsuccessful Repetition:**

- Loses balance or stumbles.
- Names less than or equal to 1 item per category for each trial.

**Performance:**

- **Trial 1:** _____________items listed Maintain good balance: YES / NO
- **Trial 2:** _____________items listed Maintain good balance: YES / NO
- **Trial 3:** _____________items listed Maintain good balance: YES / NO
- **Trial 4:** _____________items listed Maintain good balance: YES / NO
- **Trial 5:** _____________items listed Maintain good balance: YES / NO
- **Trial 6:** _____________items listed Maintain good balance: YES / NO
