# Supplementary material for: Physical therapy and deep brain stimulation in Parkinson’s Disease: protocol for a pilot randomized controlled trial
Source: Pilot Feasibility Stud. 2018 Feb 21;4:54. doi: 10.1186/s40814-018-0243-2 (PMC5822622; doi:10.1186/s40814-018-0243-2)
Supplement: Supplementary file 2 — Physical therapy for deep brain stimulation: home exercise program. (DOCX 14 kb) [file 40814_2018_243_MOESM2_ESM.docx]

**Physical Therapy for Deep Brain Stimulation**

**Home Exercise Program**

**Ryan Duncan, PT, DPT**

**Table of Contents** **Page(s)**

Trunk Rotation 3

Standing Hip Flexion 4-6

Standing Hip Abduction 7-9

Standing Plantarflexion 10-15

Standing Squat 16-21

**Home Exercise Program**

**Standing Trunk Rotation (Warm Up)**

**Level 1: Standing Trunk Rotation**

**Equipment:** Kitchen counter or sink or railing fixed to wall

Instructions: Please stand in front of the counter, sink, or railing and place both hands just above the support surface. Stand tall with your feet at a comfortable width apart. Keeping your feet in place, turn your body to the right as far as you safely can and hold for 10 seconds. Return to the starting position and hold for 10 seconds. Repeat the trunk rotation to the left side and hold for 10 seconds. Return to the starting position. Repeat this exercise so that you turn to each side 10 times. While doing this, if you feel unstable, place one hand on the support surface for safety.

**Number of Turns to Each Side: _____ / 10 on Right _____ / 10 on Left**

**Did you feel this exercise was difficult (circle one)? Y / N**

**Were you able to complete the exercise without putting your hand on the support surface (circle one)?: Y / N**

**Home Exercise Program**

**Standing Hip Flexion**

**Level 1: Standing Hip Flexion with Bilateral Hand Assist**

**Equipment:** Kitchen counter or sink or railing fixed to wall

Instructions: Please stand in front of the counter, sink, or railing and place both hands on the support surface. Stand tall and bend your hip like you are marching and hold for 2 seconds. While doing this, please remain standing tall and avoid leaning forward or to either side. Slowly bring your leg back to the starting position. Alternate this movement between right and left sides for a total of 10 repetitions on each side. Do 3 sets on each leg.

**Number of Sets Complete Per Leg: _____ / 3**

**Did you feel this exercise was difficult (circle one)? Y / N**

**Home Exercise Program**

**Standing Hip Flexion**

**Level 2: Standing Hip Flexion with Unilateral Hand Assist**

**Equipment:** Kitchen counter or sink or railing fixed to wall

Instructions: Please stand in front of the counter, sink, or railing and place only one hand on the support surface. Stand tall and bend your hip like you are marching and hold for 2 seconds. While doing this, please remain standing tall and avoid leaning forward or to either side. Slowly bring your leg back to the starting position. Alternate this movement between right and left sides for a total of 10 repetitions on each side. Do 3 sets on each leg. If you feel unstable, please place both hands on the support surface and continue the exercise.

**Number of Sets Complete Per Leg: _____ / 3 on Right _____ / 3 on Left**

**Did you feel this exercise was difficult (circle one)? Y / N**

**Were you able to complete the exercise with only one hand on the support surface (circle one)?: Y / N**

**Home Exercise Program**

**Standing Hip Flexion**

**Level 3: Standing Hip Flexion without Hand Assist**

**Equipment:** Kitchen counter or sink or railing fixed to wall

Instructions: Please stand in front of the counter, sink, or railing. Keep your hands just above the support surface, but please do not hold on if possible. Stand tall and bend your hip like you are marching and hold for 2 seconds. While doing this, please remain standing tall and avoid leaning forward or to either side. Slowly bring your leg back to the starting position. Alternate this movement between right and left sides for a total of 10 repetitions on each side. Do 3 sets on each leg. If you feel unstable, please place one hand on the support surface and continue the exercise.

**Number of Sets Complete Per Leg: _____ / 3 on Right _____ / 3 on Left**

**Did you feel this exercise was difficult (circle one)? Y / N**

**Were you able to complete the exercise without your hands on the support surface (circle one)?: Y / N**

**Home Exercise Program**

**Standing Hip Abduction**

**Level 1: Standing Hip Abduction with Bilateral Hand Assist**

**Equipment:** Kitchen counter or sink or railing fixed to wall

Instructions: Please stand in front of the counter, sink, or railing and place both hands on the support surface. Stand tall and move your leg out to the side and hold for 2 seconds. While doing this, please do not let your trunk bend to the side. Slowly bring your leg back to the starting position. Repeat this 10 times. Do 3 sets on each leg.

**Number of Sets Complete Per Leg: _____ / 3 on Right _____ / 3 on Left**

**Did you feel this exercise was difficult (circle one)? Y / N**

**Home Exercise Program**

**Standing Hip Abduction**

**Level 2: Standing Hip Abduction with Unilateral Hand Assist**

**Equipment:** Kitchen counter or sink or railing fixed to wall

Instructions: Please stand in front of the counter, sink, or railing and place only one hand on the support surface. Stand tall and move your leg out to the side and hold for 2 seconds. While doing this, please do not let your trunk bend to the side. Slowly bring your leg back to the starting position. If you feel unstable, please place both hands on the support surface and continue the exercise. Repeat this 10 times. Do 3 sets on each leg.

**Number of Sets Complete Per Leg: _____ / 3 on Right _____ / 3 on Left**

**Did you feel this exercise was difficult (circle one)? Y / N**

**Were you able to complete the exercise with only one hand on the support surface (circle one)?: Y / N**

**Home Exercise Program**

**Standing Hip Abduction**

**Level 3: Standing Hip Abduction without Hand Assist**

**Equipment:** Kitchen counter or sink or railing fixed to wall

Instructions: Please stand in front of the counter, sink, or railing. Keep your hands just above the support surface, but please do not hold on if possible. Stand tall and move your leg out to the side and hold for 2 seconds. While doing this, please do not let your trunk bend to the side. Slowly bring your leg back to the starting position. If you feel unstable, please place one hand on the support surface and continue the exercise. Repeat this 10 times. Do 3 sets on each leg.

**Number of Sets Complete Per Leg: _____ / 3 on Right _____ / 3 on Left**

**Did you feel this exercise was difficult (circle one)? Y / N**

**Were you able to complete the exercise without your hands on the support surface (circle one)**?**: Y / N**

**Home Exercise Program**

**Standing Plantarflexion (Heel Raises)**

**Level 1: Standing Bilateral Plantarflexion with Bilateral Hand Assist**

**Equipment:** Kitchen counter or sink or railing fixed to wall

Instructions: Please stand in front of the counter, sink, or railing and place both hands on the support surface. Stand tall and raise both heels as high as possible off the ground at the same time. Hold for 3 seconds. Slowly bring your heels back to the starting position. Repeat this 10 times. Do 3 sets.

**Number of Sets Complete: _____ / 3**

**Did you feel this exercise was difficult (circle one)?: Y / N**

**Home Exercise Program**

**Standing Plantarflexion (Heel Raises)**

**Level 2: Standing Bilateral Plantarflexion with Unilateral Hand Assist**

**Equipment:** Kitchen counter or sink or railing fixed to wall

Instructions: Please stand in front of the counter, sink, or railing. Place your hands just above the surface but do not hold on. Stand tall and raise both heels as high as possible off the ground at the same time. Hold for 3 seconds. Slowly bring your heels back to the starting position. During the exercise, if you feel unstable at all, grab onto the support surface. Repeat this 10 times. Do 3 sets.

**Number of Sets Complete: _____ / 3**

**Did you feel this exercise was difficult (circle one)? Y / N**

**Were you able to complete the exercise with only one hand on the support surface (circle one)?: Y / N**

**Home Exercise Program**

**Standing Plantarflexion (Heel Raises)**

**Level 3: Standing Bilateral Plantarflexion without Hand Assist**

**Equipment:** Kitchen counter or sink or railing fixed to wall

Instructions: Please stand in front of the counter, sink, or railing and place only one hand on the support surface. Stand tall and raise both heels as high as possible off the ground at the same time. Hold for 3 seconds. Slowly bring your heels back to the starting position. If you feel unstable, please place both hands on the support surface and continue the exercise. Repeat this 10 times. Do 3 sets.

**Number of Sets Complete: _____ / 3**

**Did you feel this exercise was difficult (circle one)? Y / N**

**Were you able to complete the exercise without holding on the support surface (circle one)?: Y / N**

**Home Exercise Program**

**Standing Plantarflexion (Heel Raises)**

**Level 4: Standing Unilateral Plantarflexion with Bilateral Hand Assist**

**Equipment:** Kitchen counter or sink or railing fixed to wall

Instructions: Please stand in front of the counter, sink, or railing. Place both hands on the support surface. Bend your left knee so that your foot is behind you and hold it in this position. Stand tall and raise your right heel as high as possible off the ground. Hold for 3 seconds. Slowly bring your heel back to the starting position. Repeat this 10 times. Switch legs. Do 3 sets on each leg.

**Number of Sets Complete Per Leg: _____ / 3 on Right _____ / 3 on Left**

**Did you feel this exercise was difficult (circle one)?: Y / N**

**Home Exercise Program**

**Standing Plantarflexion (Heel Raises)**

**Level 5: Standing Unilateral Plantarflexion with Unilateral Hand Assist**

**Equipment:** Kitchen counter or sink or railing fixed to wall

Instructions: Please stand in front of the counter, sink, or railing. Place one hand on the support surface. Bend your left knee so that your foot is behind you and hold it in this position. Stand tall and raise your right heel as high as possible off the ground. Hold for 3 seconds. Slowly bring your heel back to the starting position. If you feel unstable at all throughout the exercise, put both hands on the support surface. Repeat this 10 times. Switch legs. Do 3 sets on each leg.

**Number of Sets Complete Per Leg: _____ / 3 on Right _____ / 3 on Left**

**Did you feel this exercise was difficult (circle one)? Y / N**

**Were you able to complete the exercise with only one hand on the support surface (circle one)?: Y / N**

**Home Exercise Program**

**Standing Plantarflexion (Heel Raises)**

**Level 6: Standing Unilateral Plantarflexion without Hand Assist**

**Equipment:** Kitchen counter or sink or railing fixed to wall

Instructions: Please stand in front of the counter, sink, or railing. Keep your hands just above the support surface. Bend your left knee so that your foot is behind you and hold it in this position. Stand tall and raise your right heel as high as possible off the ground. Hold for 3 seconds. Slowly bring your heel back to the starting position. If you feel unstable at all throughout the exercise, put both one or both hands on the support surface. Repeat this 10 times. Switch legs. Do 3 sets on each leg.

**Number of Sets Complete Per Leg: _____ / 3 on Right _____ / 3 on Left**

**Did you feel this exercise was difficult (circle one)? Y / N**

**Were you able to complete the exercise without holding on the support surface (circle one)?: Y / N**

**Home Exercise Program**

**Standing Squat**

**Level 1: Standing Squat with Bilateral Hand Assist**

**Equipment:** Kitchen counter or sink or railing fixed to wall

Instructions: Please stand in front of the counter, sink, or railing and place both hands on the support surface. Bend your hips and knees slowly as if you were going to sit halfway to a chair. Hold for 3 seconds at the halfway point. Then return to the starting position while squeezing your buttocks. Repeat this 10 times. Do 3 sets on each leg.

**Number of Sets Complete: _____ / 3**

**Did you feel this exercise was difficult (circle one)?: Y / N**

**Home Exercise Program**

**Standing Squat**

**Level 2: Standing Squat with Unilateral Hand Assist**

**Equipment:** Kitchen counter or sink or railing fixed to wall

Instructions: Please stand in front of the counter, sink, or railing. Place one hand on the support surface. Bend your hips and knees slowly as if you were going to sit halfway to a chair. Hold for 3 seconds at the halfway point. Then return to the starting position while squeezing your buttocks. If you feel unstable at all throughout the exercise, place both hands on the support surface. Repeat this 10 times. Do 3 sets on each leg.

**Number of Sets Complete Per Leg: _____ / 3 on Right _____ / 3 on Left**

**Did you feel this exercise was difficult (circle one)? Y / N**

**Were you able to complete the exercise with only one hand on the support surface (circle one)?: Y / N**

**Home Exercise Program**

**Standing Squat**

**Level 3: Standing Squat without Hand Assist**

**Equipment:** Kitchen counter or sink or railing fixed to wall

Instructions: Please stand in front of the counter, sink, or railing. Place your hands just above support surface. Bend your hips and knees slowly as if you were going to sit halfway to a chair. Hold for 3 seconds at the halfway point. Then return to the starting position while squeezing your buttocks. If you feel unstable at all throughout the exercise, place one hand on the support surface. Repeat this 10 times. Do 3 sets on each leg.

**Number of Sets Complete Per Leg: _____ / 3**

**Did you feel this exercise was difficult (circle one)? Y / N**

**Were you able to complete the exercise without your hands on the support surface (circle one)?: Y / N**

**Home Exercise Program**

**Standing Squat**

**Level 4: Single Leg Squat with Bilateral Hand Assist**

**Equipment:** Kitchen counter or sink or railing fixed to wall

Instructions: Please stand in front of the counter, sink, or railing and place both hands on the support surface. Bend your left knee so that your foot is behind you. While standing on your right leg, bend your hip and knee slowly as if you were going to sit halfway to a chair. Hold for 3 seconds at the halfway point. Then return to the starting position while squeezing your buttocks. Repeat this 10 times. Do 3 sets on each leg.

**Number of Sets Complete: _____ / 3**

**Did you feel this exercise was difficult (circle one)?: Y / N**

**Home Exercise Program**

**Standing Squat**

**Level 5: Single Leg Squat with Unilateral Hand Assist**

**Equipment:** Kitchen counter or sink or railing fixed to wall

Instructions: Please stand in front of the counter, sink, or railing and place one hand on the support surface. Bend your left knee so that your foot is behind you. While standing on your right leg, bend your hip and knee slowly as if you were going to sit halfway to a chair. Hold for 3 seconds at the halfway point. Then return to the starting position while squeezing your buttocks. If you feel unstable at all throughout the exercise, place both hands on the support surface. Repeat this 10 times. Do 3 sets on each leg.

**Number of Sets Complete: _____ / 3**

**Did you feel this exercise was difficult (circle one)? Y / N**

**Were you able to complete the exercise with only one hand on the support surface (circle one)?: Y / N**

**Home Exercise Program**

**Standing Squat**

**Level 6: Single Leg Squat without Hand Assist**

**Equipment:** Kitchen counter or sink or railing fixed to wall

Instructions: Please stand in front of the counter, sink, or railing. Keep your hands just above the support surface. Bend your left knee so that your foot is behind you. While standing on your right leg, bend your hip and knee slowly as if you were going to sit halfway to a chair. Hold for 3 seconds at the halfway point. Then return to the starting position while squeezing your buttocks. If you feel unstable at all throughout the exercise, place one hand on the support surface. Repeat this 10 times. Do 3 sets on each leg.

**Number of Sets Complete: _____ / 3**

**Did you feel this exercise was difficult (circle one)? Y / N**

**Were you able to complete the exercise without your hands on the support surface (circle one)?: Y / N**
